# Directed Evolution of (*R*)-2-Hydroxyglutarate Dehydrogenase Improves 2-Oxoadipate Reduction by 2 Orders of Magnitude

**DOI:** 10.1021/acssynbio.2c00162

**Published:** 2022-08-08

**Authors:** Veronica Saez-Jimenez, Simone Scrima, Matteo Lambrughi, Elena Papaleo, Valeria Mapelli, Martin K. M. Engqvist, Lisbeth Olsson

**Affiliations:** † Division of Industrial Biotechnology, Department of Biology and Biological Engineering, Chalmers University of Technology, 412 96 Gothenburg, Sweden; ‡ Cancer Structural Biology, Danish Cancer Society Research Center, 2100 Copenhagen, Denmark; § Cancer Systems Biology, Section for Bioinformatics, Department of Health and Technology, Technical University of Denmark, 2800 Lyngby, Denmark; ∥ Division of Systems and Synthetic Biology, Department of Biology and Biological Engineering, Chalmers University of Technology, 412 96 Gothenburg, Sweden

**Keywords:** adipic acid, protein engineering, random mutagenesis, saturation mutagenesis, (*R*)-2-hydroxyacid dehydrogenase, (*R*)-2-hydroxyadipate

## Abstract

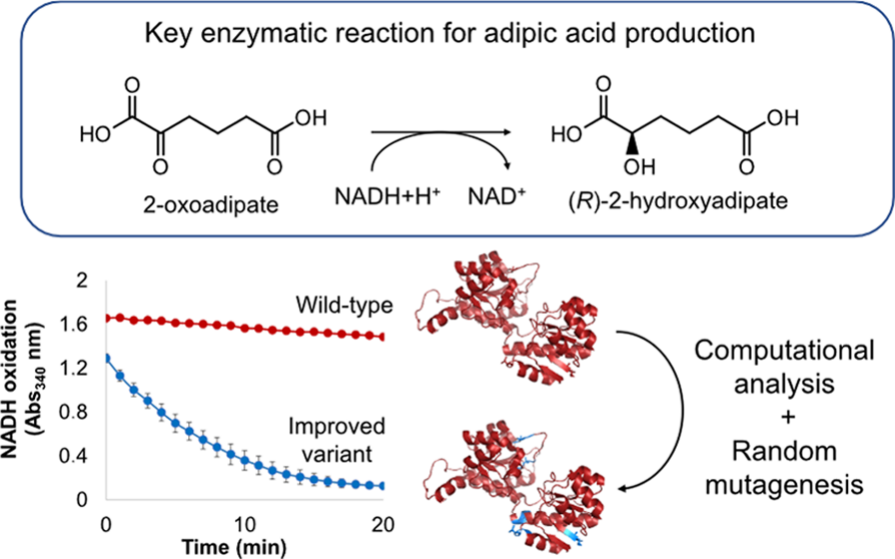

Pathway engineering is commonly employed to improve the production of various metabolites but may incur in bottlenecks due to the low catalytic activity of a particular reaction step. The reduction of 2-oxoadipate to (*R*)-2-hydroxyadipate is a key reaction in metabolic pathways that exploit 2-oxoadipate conversion via α-reduction to produce adipic acid, an industrially important platform chemical. Here, we engineered (*R*)-2-hydroxyglutarate dehydrogenase from *Acidaminococcus fermentans* (Hgdh) with the aim of improving 2-oxoadipate reduction. Using a combination of computational analysis, saturation mutagenesis, and random mutagenesis, three mutant variants with a 100-fold higher catalytic efficiency were obtained. As revealed by rational analysis of the mutations found in the variants, this improvement could be ascribed to a general synergistic effect where mutation A206V played a key role since it boosted the enzyme’s activity by 4.8-fold. The Hgdh variants with increased activity toward 2-oxoadipate generated within this study pave the way for the bio-based production of adipic acid.

## Introduction

Adipic acid is a key intermediate in chemical production, as it is used extensively in the nylon industry. The International Energy Agency regards it as the most important industrial dicarboxylic acid, with a market value of almost 6.3 billion USD.^1,2^ At present, adipic acid is produced from fossil-based, nonrenewable materials, through a process that releases N_2_O, a very potent greenhouse gas. It is estimated that around 10% of the N_2_O released into the atmosphere comes from the production of adipic acid; hence, replacing the current process with a bio-based one that employs renewable precursors would greatly reduce greenhouse gas emissions.^3−5^

**Figure 1:**
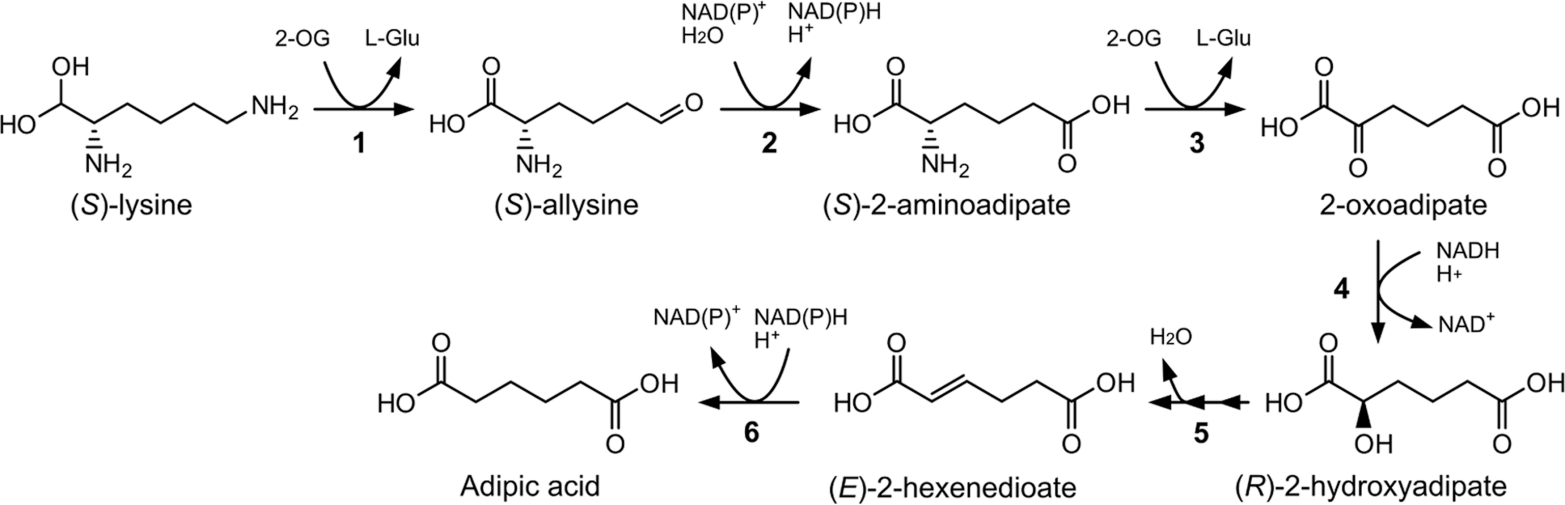
Metabolic pathway for the production of adipic acid from (*S*)-lysine. The conversion of 2-oxoadipate to (*R*)-2-hydroxyadipate (reaction 4) is a key bottleneck in this pathway and the focus of this study. Reaction 1 is performed by an enzyme corresponding to E.C. 2.6.1.36, reaction 2 is catalyzed by an enzyme E.C. 1.2.1.31, reaction 3 is catalyzed by an enzyme E.C. 2.6.1.39, and reaction 6 is catalyzed by an enzyme with activity E.C.1.3.1.31. The direct conversion of (*R*)-2-hydroxyadipate to (*E*)-2-hexenedioate (reaction 5) has not been demonstrated. It is possible through the activation to 2-hydroxyadipyl-CoA, dehydration to 5-carboxy-2-pentenoyl-CoA, and, finally, the release of coenzyme A to yield (*E*)-2-hexenedioate. Abbreviations: 2-oxoglutarate (2-OG); l-glutamate (l-Glu).

The biological synthesis of adipic acid has become a hot topic in the past years and a number of metabolic pathways have been suggested. While some of them have been experimentally evaluated, titers and yields remain low and do not meet industrial requirements.^2,5,6^ One of the main problems is an unbalanced gene expression or the low activity of the enzymes participating in the pathway, which leads to the accumulation of metabolic intermediates and, consequently, low yields.^2,6^ One of these metabolic pathways (Figure 1) starts with (*S*)-lysine (or l-lysine),^7,8^ an essential metabolite, which can be produced from glucose using microbial cell factories.^9^ A constraint to the implementation of this route is the low activity of 2-oxoadipate reduction (Figure 1, reaction 4).^10,11^ Finding an enzyme with elevated catalytic activity toward 2-oxoadipate would strongly benefit bio-based production of adipic acid through this and other metabolic pathways that exploit the α-reduction of 2-oxoadipate.^6,10,12^

Enzymes belonging to the (*R*)-isomer-specific 2-hydroxyacid dehydrogenase family have been described to catalyze 2-oxoadipate reduction, but with low rates or specificity; they include the (*R*)-2-hydroxyglutarate dehydrogenase from *Acidaminococcus fermentans* (Hgdh, Uniprot code D2RJU7)^10^ and an engineered variant of the homoisocitrate dehydrogenase from *Schizosaccharomyces pombe*, whose catalytic efficiency was only 13.3 M^–1^ s^–1^^11^ (*K*_m_ for 2-oxoadipate was 2.0 mM, and *k*_cat_ was 1.6 min^–1^). Furthermore, a lactate dehydrogenase from *Alcaligenes eutrophus* has been described to reduce 2-oxoadipate for adipic acid production.^13^ However, this enzyme belongs to the evolutionary distinct family of (l)-2-hydroxyacid dehydrogenases and produces (*S*)-2-hydroxyadipate instead of its (*R*)-enantiomer (Figure 1, reaction 4) that is required to produce adipic acid in the proposed metabolic pathway.^10,14^

**Figure 2:**
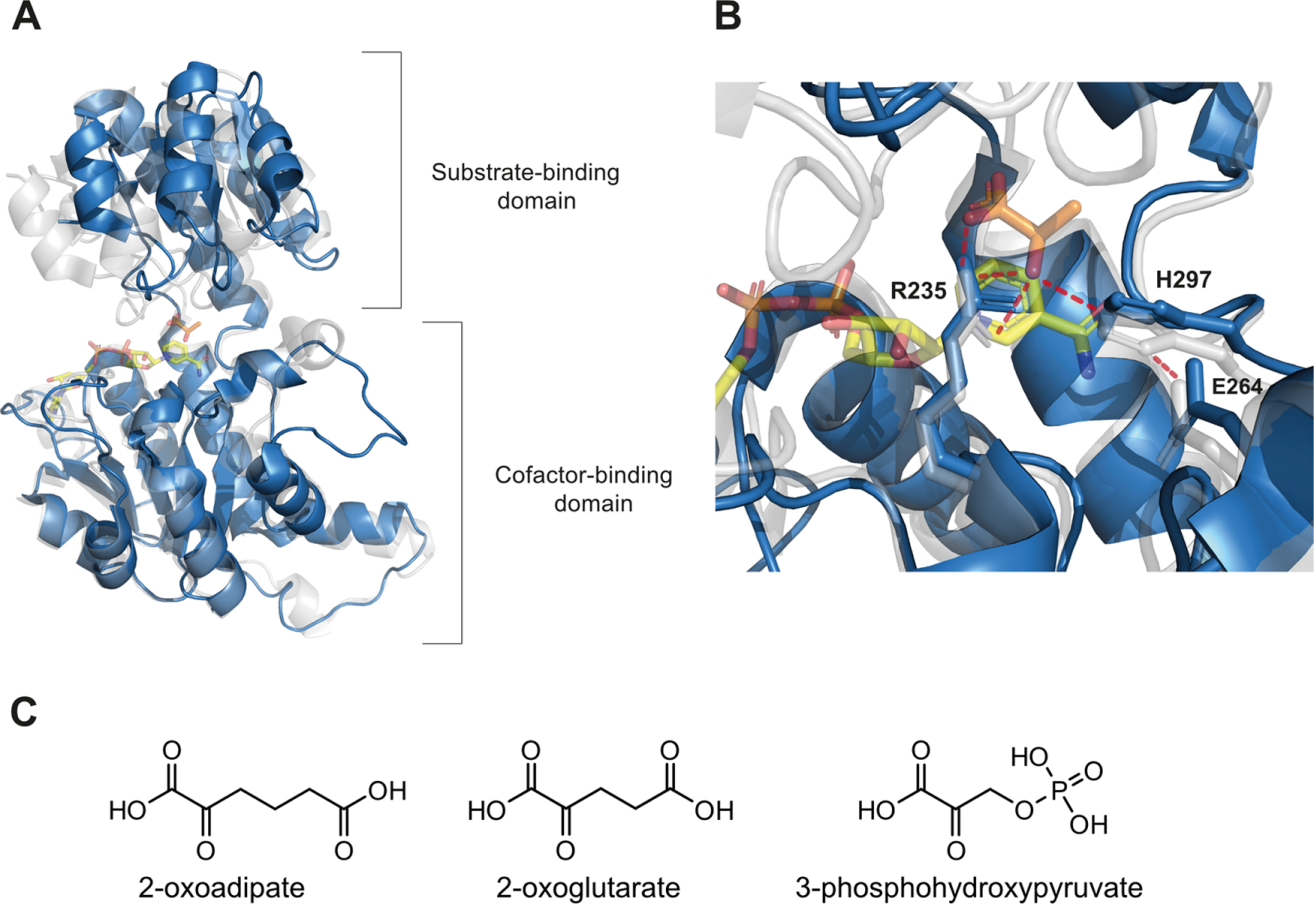
Structure of (*R*)-2-hydroxyacid dehydrogenase family members, key residues involved in substrate catalysis, and substrates of Hgdh and Pgdh. (A) Monomer of (*R*)-2-hydroxyglutarate dehydrogenase (Hgdh from *A. fermentans*, PDB ID 1XDW) in the open conformation (blue), structurally aligned with a monomer of d-lactate dehydrogenase (LDH from *Aquifex aeolicus*, PDB ID 3KB6) in the closed conformation (transparent white). The two enzymes were aligned based on the cofactor-binding domain. The NADH cofactor (yellow) and lactate (orange) substrate of LDH are shown. (B) Active site of both enzymes showing the substrate (orange) and NADH (yellow) in the active site of LDH, as well as the catalytic residues numbered following the Hgdh amino acid sequence. Red dashed lines show the interactions between the side chains of the catalytic triad of LDH and the lactate substrate. (C) Chemical structures of 2-oxoadipate (substrate of the reaction studied in this work), 2-oxoglutarate (natural substrate of Hgdh and Pgdh), and 3-phosphohydroxypyruvate (natural substrate of Pgdh).

Members of the (*R*)-2-hydroxyacid dehydrogenase family can reversibly catalyze the reduction of 2-oxo acids to the corresponding 2-hydroxy acids, with simultaneous oxidation of the cofactors NADH or NADPH. Usually, these enzymes exist as homodimers, with each monomer composed of a cofactor-binding domain and a substrate-binding domain (Figure 2A). Both domains show structural variants of the βαβ Rossmann fold. The active site is located in a cleft between the two domains where, in the absence of cofactor and substrate, it is exposed to the solvent. Binding of the cofactor promotes the transition to the closed conformation, which is necessary for the formation of a working active site and catalysis. Binding of the substrate to residues from both domains is thought to shift the equilibrium toward the closed conformation.^15−18^

The catalytic triad (R235, E264, and H297, according to Hgdh amino acid sequence numbering, Figure 2B) is conserved among the different members of the (*R*)-2-hydroxyacid dehydrogenase family. Specifically, H297 acts as an internal acid-base catalyst during the reduction reaction, whereby a hydride anion is transferred from the cofactor to the substrate and a proton is transferred to the carbonyl oxygen of the substrate. The residue R235 mediates substrate recognition and orientation as it binds to the α-carboxylate, as well as the polarization of the carbonyl group of the substrate, increasing its susceptibility to a nucleophilic attack. In addition, E264, which is bound to H297 through a hydrogen bond (Figure 2B), plays a critical role in orienting the imidazole ring of the positively charged H297, thus stabilizing its protonated state and facilitating the proton transfer reaction.^15−17,19^

**Figure 3:**
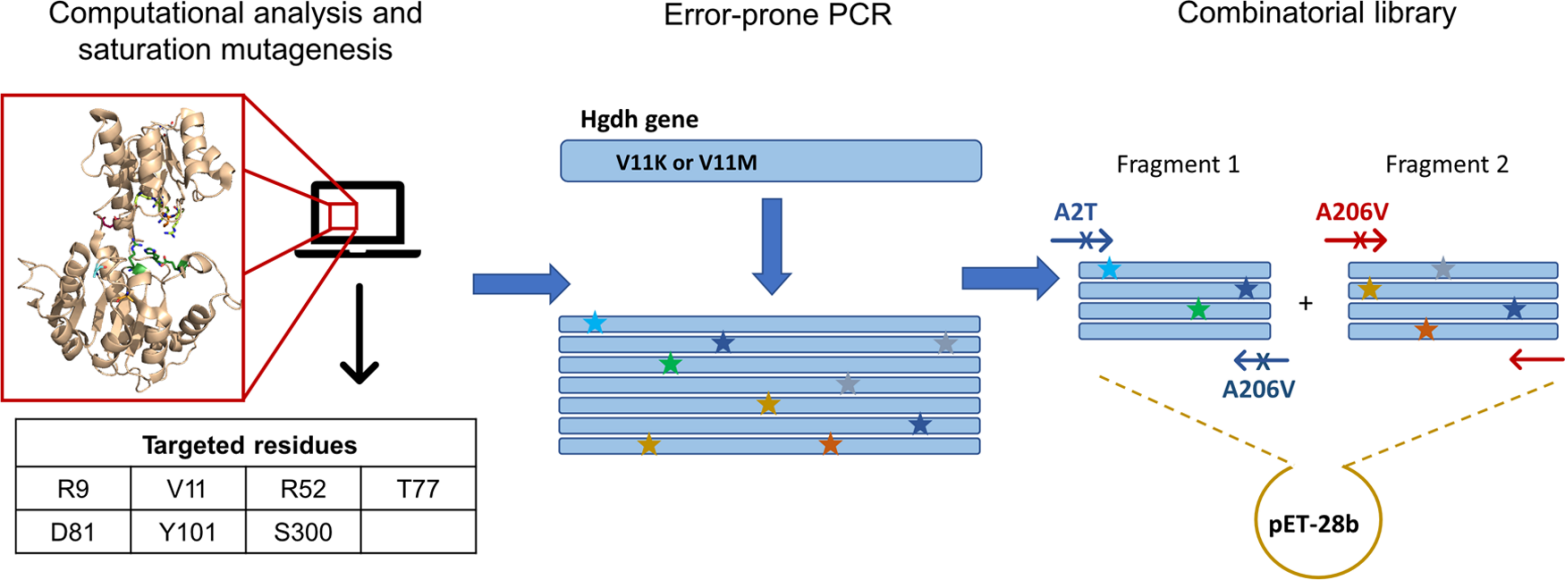
Protein engineering strategies and workflow employed in the present study. A computational analysis combining the evolution-guided approach of FuncLib^20^ and in silico high-throughput saturation mutagenesis^21,22^ was carried out to design mutant variants that could improve 2-oxoadipate activity. Seven residues were targeted for saturation mutagenesis. Subsequently, a round of whole-gene random mutagenesis (error-prone PCR) was carried out using as template the best variants found in the previous round. Finally, a combinatorial library that mixed some of the potentially beneficial mutations found by error-prone PCR was designed and screened.

Given that no efficient enzyme for the production of (*R*)-2-hydroxyadipate has been described so far, the aim of the present study was to identify suitable target enzymes for improvement via directed evolution. Two candidate enzymes, that is, Hgdh from *A. fermentans* and d-3-phosphoglycerate dehydrogenase from *Escherichia coli* (Pgdh), were selected due to the structural similarity of their natural substrates to 2-oxoadipate (Figure 2C). After assessing 2-oxoadipate reduction by the two enzymes, Hgdh was identified as the one with the highest activity. Hgdh was then evolved using a combination of computational analysis and protein engineering (Figure 3), which led to the discovery of three enzyme variants with a 100-fold higher catalytic efficiency. Finally, a rational analysis was carried out to identify the mutations responsible for improving the enzyme’s activity.

## Results and Discussion

### Two Candidate Dehydrogenases Can Successfully Reduce 2-Oxoadipate

Two enzymes from the (*R*)-2-hydroxyacid dehydrogenase family were selected, and their catalytic activity toward 2-oxoadipate was measured. The first enzyme was d-3-phosphoglycerate dehydrogenase from *E. coli* (Pgdh, E.C. 1.1.1.95), which was selected due to the structural similarity of its natural substrate, 2-oxoglutarate,^23,24^ to 2-oxoadipate (Figure 2C). In addition, this enzyme is also able to catalyze the reduction of 3-phosphohydroxypyruvate (Figure 2C), even though the 2-oxoglutarate and 3-phosphohydroxypyruvate show some structural differences; the former showing a γ-carboxylate and the latter a phosphate group. This fact suggests that Pgdh might accept other structurally related substrates such as 2-oxoadipate. The second selected enzyme was Hgdh from *A. fermentans* (E.C. 1.1.99.39), which catalyzes the reduction of 2-oxoglutarate and, to a much lesser extent, 2-oxoadipate.^10^

To assess the activity of Pgdh and Hgdh toward 2-oxoadipate and 2-oxoglutarate, both enzymes were heterologously expressed in *E. coli* and then purified (Figure S1). The kinetic constants were determined (Table 1 and Figure S2).

**Table 1:** Kinetic Constants for the Reduction of 2-Oxoadipate and 2-Oxoglutarate by Hgdh and Pgdh

substrate	enzyme	*k*_cat_ (s^–1^)	*K*_m_ (mM)	*k*_cat_/*K*_m_ (M^–1^ s^–1^)
2-oxoadipate	Hgdh^a^	0.70 ± 0.03	2.96 ± 0.24	2.4 × 10^2^
	Pgdh^b^	0.27 ± 0.02	5.10 ± 0.63	5.3 × 10
2-oxoglutarate	Hgdh^a^	1223 ± 24	0.33 ± 0.01	3.7 × 10^6^
	Pgdh^b^	2.02 ± 0.11	0.084 ± 0.017	2.4 × 10^4^

a Reactions with Hgdh were carried out at 25 °C in 50 mM phosphate buffer, pH 8, containing 0.25 mM NADH.

b Reactions with Pgdh were carried out at 37 °C in 50 mM phosphate buffer, pH 7.4, containing 0.25 mM NADH and 1 mM DTT. Pgdh activity with 2-oxoadipate was observed also at 25 **°**C, but it was too low to determine the kinetic constants. The activity of Pgdh observed at 37 °C demonstrates that the enzyme was active and properly folded. Results are expressed as the mean ± standard deviation of three replicate assays.

Even though the kinetic parameters of Pgdh were significantly lower when using 2-oxoadipate instead of 2-oxoglutarate as substrate (Table 1), the enzyme was nevertheless capable of reducing 2-oxoadipate. The catalytic constant (*k*_cat_) of Pgdh for 2-oxoadipate was 7.5-fold lower than the *k*_cat_ for 2-oxoglutarate. Likewise, the observed Michaelis constant (*K*_m_) was 61-fold higher for 2-oxoadipate than for 2-oxoglutarate. Overall, the efficiency of Pgdh was 450-fold lower with 2-oxoadipate than with 2-oxoglutarate. Hgdh was active toward both 2-oxoglutarate and 2-oxoadipate (Table 1), in agreement with previous observations by Parthasarathy et al.^10^ Again, the reduction of 2-oxoadipate was not as efficient as that of 2-oxoglutarate, as indicated by a 1750-fold lower *k*_cat_ and a 9 times higher *K*_m_ for the former compared to the latter. Overall, Hgdh exhibited a 15 000-fold lower catalytic efficiency with 2-oxoadipate than with 2-oxoglutarate.

These results indicate that the catalytic pockets of both Pgdh and Hgdh can be adapted to catalyze the reduction of 2-oxoadipate even if with a low efficiency. This finding is not surprising given that some (*R*)-2-hydroxyacid dehydrogenases are considered promiscuous and can accept a wide range of substrates.^15^ Notably, even though the backbone of 2-oxoadipate is only one carbon longer than that of 2-oxoglutarate (Figure 2C), the substrates maintain the same physicochemical properties dictated largely by the two terminal carboxylate groups.

Hgdh was selected as the most suitable engineering target as its reduction of 2-oxoadipate was more efficient compared to that by Pgdh (Table 1). Moreover, Hgdh showed good stability, which is desirable for protein engineering, and the enzyme’s activity was easily monitored at room temperature, whereas Pgdh activity was only measurable at 37 °C.

### Computational Studies and Saturation Mutagenesis Experiments: V11M and V11K Mutations Improve Activity toward 2-Oxoadipate

**Figure 4:**
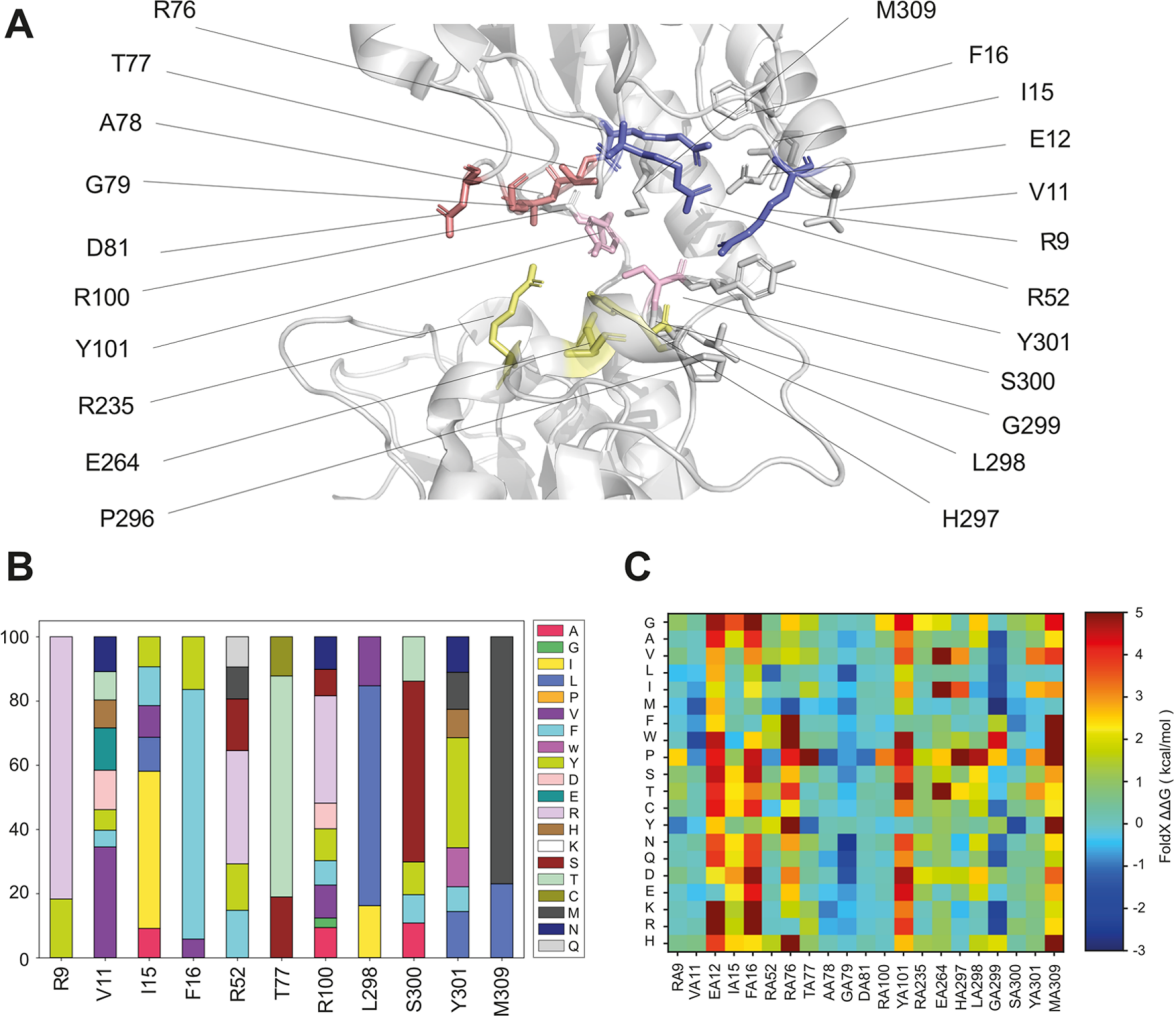
Computational analyses. (A) Representation of the Hgdh active site pocket. The residues included in the FuncLib scans are represented by sticks: (i) residues in the polar pocket responsible for the binding of the α-carboxylate of the substrate are shown in red, (ii) residues of the polar patch responsible for orienting the substrate in the binding pocket are shown in pink, (iii) residues of the arginine cluster are shown in blue, (iv) other residues are shown in gray. The residues of the catalytic triad are highlighted in yellow. (B) Barplot showing the percentage of mutant variants at each selected amino acid position in the ensemble of multipoint mutants from the third FuncLib calculation. (C) Heatmap showing the ΔΔ*GS* of folding between the mutated variants and wild-type Hgdh for each possible mutation (*y*-axis) at the selected amino acid position (*x*-axis) calculated using FoldX.

As a first approach in the protein engineering of Hgdh, a combination of computational studies and saturation mutagenesis was used to find beneficial mutations capable of improving 2-oxoadipate reduction activity. Specifically, the evolution-guided approach of FuncLib, based on phylogenetic analysis and Rosetta design calculations,^20^ was used to design and rank mutated variants of Hgdh at multiple sites that could increase activity toward 2-oxoadipate. It has been shown that FuncLib is effective in designing ensembles of stable and functionally diverse multipoint mutants of enzymes.^20^ We aimed to identify amino acid positions that are predicted by FuncLib to have high variability of allowed mutations (Figure 4B) in the calculated ensembles of multipoint mutants of Hgdh to prioritize them for the experimental engineering strategies. Three mutational screenings were performed, each including different amino acid positions free to mutate during the FuncLib calculations, considering a total of 19 positions (for additional details, see the Methods section, Figures 4A,B, and S3). We selected these residues from the ones forming the first (R9, R52, R76, T77, A78, G79, D81, Y101, and S300) and second (V11, E12, I15, F16, R100, P296, L298, G299, Y301, and M309) shell of the Hgdh active site (Figure 4A). In addition, we included residues proposed to be involved in the binding of 2-oxoglutarate.^19^ We complemented the FuncLib scans with in silico saturation mutagenesis using MutateX, an automated pipeline^22^ based on the FoldX energy function.^21^ It has been shown that FoldX is effective in predicting destabilizing mutations in proteins. We used FoldX with the aim to estimate the potential impact of the mutations predicted by FuncLib on the thermodynamic stability of Hgdh, calculated as changes in free energy upon mutations (ΔΔ*G* values, Figure 4C), and avoid including the mutations predicted with destabilizing effects, i.e., high ΔΔ*G* values, in the experimental engineering strategies.

Overall, the three FuncLib scans identified similar mutant variants at each amino acid position (Figures 4B and S3). Low mutational variability characterized sites E12, F16, R76–A78, D81, Y101, L298, G299, and M309, resulting in conserved mutations or none at all, which ensured similar physicochemical properties for multipoint mutants (Figure 4B). For positions E12, F16, R76, G79, Y101, and M309, FoldX predicted an elevated average ΔΔ*G* upon mutation (Figure 4C), suggesting that nonconservative substitutions could affect the structural stability of Hgdh. In contrast, T77, A78, and D81 displayed only close to zero or negative ΔΔ*G* values (Figure 4C).

A greater variability of allowed mutations was observed for residues R9, V11, R52, R100, S300, and Y301, with FoldX predicting negative or close to zero ΔΔ*G* values (Figures 4B,C, and S3). It was previously suggested that R52, T77, A78, D81, and S300 were involved in determining substrate specificity of 2-hydroxyacid dehydrogenases.^15−17,19^ Considering our results obtained using FuncLib, FoldX, as well as already published data, eight residues were selected for saturation mutagenesis: R9, V11, R52, T77, A78, D81, Y101, and S300.

Saturation mutagenesis was carried out on the selected residues. The high-throughput screening assay used to measure the variants’ activity had a coefficient of variance (CV) of 14.1% (Figure S4), which ensured the reliability of the assay. Variants with improved activity (activity higher than the average activity of the parent strain plus two standard deviations) were found in library V11. Specifically, variants V11K and V11M showed 64 and 70% greater activity, respectively, compared to wild-type Hgdh, when 2 mM 2-oxoadipate was used as substrate. To determine if the increased activity was observed at lower substrate concentrations, 0.5 mM 2-oxoadipate was used, which resulted in 31% (V11K) and 39% (V11M) higher activity. The residue V11 is located in the vicinity of residues that participate in the binding and orientation of the substrate that are E12 and I15 (part of a polar patch and a hydrophobic patch defining the active site), and R9 (part of the arginine cluster binding the ω-carboxylate of the substrate) (Figure 4A). Moreover, V11 is close to Y301 and F140′ (from the second monomer), which also define the active site. Mutations V11K and V11M can lead to subtle changes in the positioning of these residues, improving the accommodation of 2-oxoadipate and, therefore, increasing activity.

The libraries generated via saturation mutagenesis of the remaining residues did not yield improved activities. In fact, some of the libraries showed a very low percentage of functional variants, whose activity toward 2-oxoadipate was higher than the average activity of wild-type Hgdh minus two standard deviations (Table S1). Specifically, mutations at R52, T77, S300, and R9 displayed only 2, 4, 8, and 10% of functional variants, respectively. These results suggest that these residues play a fundamental role in Hgdh functioning, either structurally or catalytically. R9 and R52 have been shown to form a positively charged patch (together with R76) involved in substrate recognition and binding of the γ-carboxylate group of 2-oxoglutarate. While R9 and R76 are conserved among D-2-hydroxyacid dehydrogenases, R52 is highly variable and has been suggested to modulate substrate specificity of the members of this family.^15,19^ In this case, any change to R52 was detrimental to Hgdh activity, with only 2% of variants being as active as wild-type Hgdh. It is likely that the positively charged side chain of arginine is essential for the binding and orientation of 2-oxoadipate.

Similar to R52, only 4% of the variants mutated at T77 were functional. Instead, a higher fraction of functional variants (16%) was obtained in the D81 library. Residues T77 and D81, together with A78 and G79, form a polar patch involved in the binding of the substrate’s α-carboxylate to A78 and G79 via hydrogen bonds. Mutations in T77 and D81 could easily affect the positioning of residues within the patch, thereby affecting the binding and proper orientation of the substrate in the catalytic pocket. This can explain the low percentage of functional colonies found in these libraries. Similarly, even though a relatively high mutagenic variability was found in our computational results for position S300 (Table S1), this residue is involved (together with Y101) in defining the active pocket dimension and substrate specificity; therefore, mutations at this site can interfere with proper orientation of the substrate.

### Error-Prone PCR Generates Variants with Improved Activity

**Figure 5:**
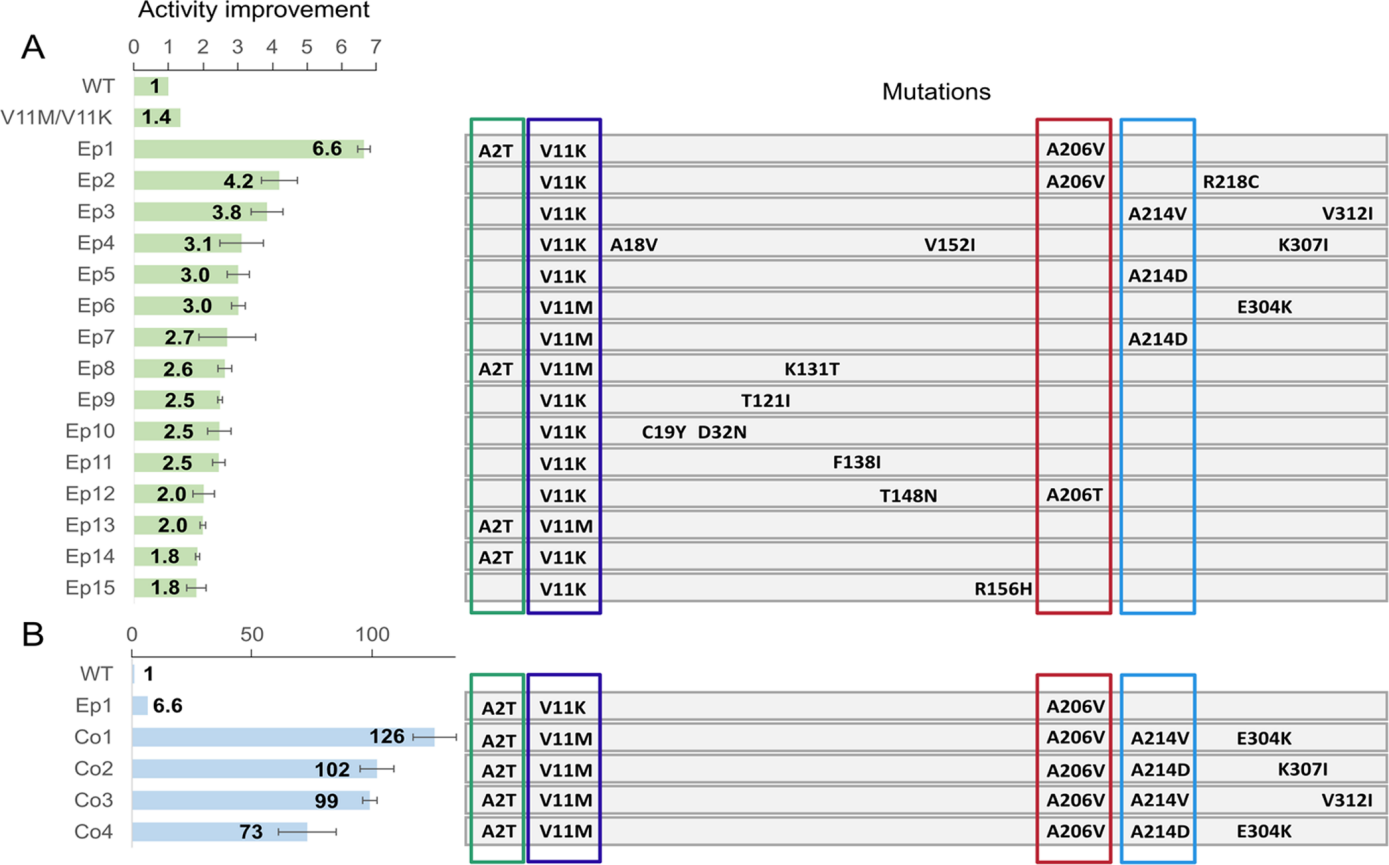
Clones with improved activity toward 2-oxoadipate obtained from the ep-PCR and combinatorial library. (A) Improved variants obtained by ep-PCR. (B) Improved variants obtained in the combinatorial library. The fold improvement measured for each variant, as well as the mutations in each of them, is shown. The average activity of wild-type Hgdh was normalized to 1 and used as reference to determine the activity of the clones analyzed. Improved variants obtained by ep-PCR were named as “Ep” followed by a number indicating the ranking based on the level of activity observed. Improved variants obtained from the combinatorial library were named as “Co” plus a number indicating the ranking based on the level of activity detected. Reactions were carried out at 25 °C in 50 mM phosphate buffer, pH 8, containing 0.5 mM 2-oxoadipate, 0.25 mM NADH, and an appropriate aliquot of culture lysate.

Even though the Hgdh V11M and V11K variants obtained showed improved activity, our aim was to further increase the ability of the enzyme to reduce 2-oxoadipate. To this end, a round of whole-gene error-prone PCR (ep-PCR) was performed using Hgdh V11M and Hgdh V11K as parent strains. The goal was to find Hgdh variants with higher catalytic efficiency (*k*_cat_/*K*_m_). To make sure that mutant variants with low *K*_m_ (as well as improved *k*_cat_) were found, substrate concentrations lower than the *K*_m_ for 2-oxoadipate were used in the screenings of libraries. For this reason, in this and the following experiments, the substrate concentration was lowered further to 0.5 mM. The screening of the obtained library revealed fifteen clones with improved activity toward 2-oxoadipate (Figure 5A).

The best variant obtained in this round (Ep1) showed 4.9 times higher activity than the parental V11M and V11K mutants, and 6.6-fold more activity compared to wild-type Hgdh. This clone contained three mutations: A2T, V11K, and A206V. Interestingly, the latter appeared also in the second-best clone (Ep2, V11K/A206V/R218C), which showed a 3.1-fold improvement compared to the parent variants. In clone Ep12 (V11K/T148N/A206T), which showed around 1.5-fold higher activity compared to the parental gene, A206 was mutated to threonine. Notably, mutation A2T occurred also in other clones showing improved activity. It is worth mentioning that the alanine in position 2 was a result of the cloning of the gene and was not initially present in Hgdh sequence. When A2T was combined with V11M or V11K, a 1.4-fold (Ep13) or 1.3-fold (Ep14) improvement was observed compared to the parent enzymesHgdh-V11M and Hgdh-V11K.

Mutations of residue A214 to valine or aspartate were also detected in clones with improved activity. In particular, clone Ep3 (V11K/A214V/V312I) displayed 2.8-fold higher activity compared to the parent enzymes V11K and V11M, whereas clones Ep5 (V11K/A214D) and Ep7 (V11M/A214D) displayed a 2.2- and 2.0-fold increment, respectively. These results indicate that A214D has a clear positive effect on the observed activity. Interestingly, mutation E304K (present in clone Ep6 together with V11M) also showed a positive effect, with a 2.2-fold improvement compared to V11M alone.

### Combinatorial Library Generates Variants Whose Activity Is Improved by 2 Orders of Magnitude

Given the many clones with improved activity created by the ep-PCR library, we decided to investigate if a combination of these potentially beneficial mutations could have an additive effect and further improve the activity of Hgdh toward 2-oxoadipate. To this end, a combinatorial library that mixed the mutations present in the improved variants was generated. The library was designed in such a way that all of the clones contained: (i) mutations V11M or V11K derived from saturation mutagenesis of V11; (ii) mutations A2T and A206V found in the best clone (Ep1) from the ep-PCR library; and (iii) some other mutations found in the best clones from the ep-PCR library (Table S2). To construct this library, the *hgdh* gene was amplified as two different fragments. The first fragment contained the coding sequence corresponding to residues 1–206 and was amplified using plasmid templates that included mutations A18V and V152I, C19Y and D32N, as well as T121I, K131T, F138I, T148N, or R156H (Table S2). The second fragment contained the coding sequence corresponding to residues 206 to 346 and was amplified using plasmids that contained mutations A214V and V312I, A214D, R218C, or K307I, and E304K (Table S2). The pools of the two fragments were then combined with the vector, giving rise to 63 possible distinct variants.

Screening of the library revealed several variants, whose activity was between 11.1- and 19.1-fold higher compared to that of the parent Ep1 clone (A2T, V11K, A206V) (Figure 5B). Overall, the best variant (Co1) displayed a 126-fold increment in activity compared to wild-type Hgdh; it combined the mutations A2T, V11M, and A206V with A214V and E304K. Notably, all improved variants included mutation A214D or A214V, together with A2T, V11M, and A206V. Some unexpected combinations were also found in the library, such as A214V and E304K (Co1), A214D and K307I (Co2), and A214D and E304K (Co4). Ambiguous is how these combinations were combined. The most plausible explanation may have to do with the generation of primers A206V_D and A206V_R (Table S3) used in the PCR. These primers contained the DNA region encoding for residues D200 to G213. The inclusion of some unintended extra nucleotides (encoding for A214D or A214V) could explain the encountered combination of mutations.

### Effect of Single Mutations on Activity toward 2-Oxoadipate and 2-Oxoglutarate

**Figure 6:**
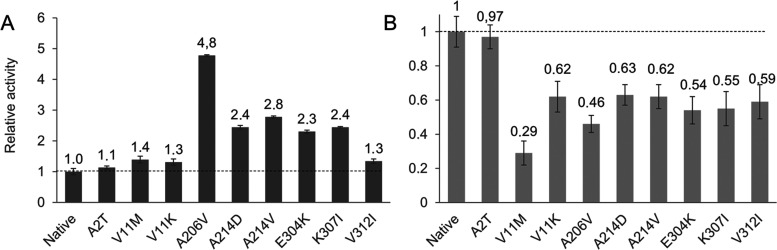
Activity of single mutant variants. (A) Activity of the variants toward 2-oxoadipate. (B) Activity of the variants toward 2-oxoglutarate. Reactions were carried out at 25 °C in 50 mM phosphate buffer, pH 8, containing 0.5 mM 2-oxoadipate or 2-oxoglutarate, 0.25 mM NADH, and an appropriate aliquot of culture lysate. Results are expressed as the mean ± standard deviation of replicate assays.

The most improved variants found upon construction of the libraries contained several amino acid substitutions (Figure 5B). To understand which of the mutations actually contributed to higher catalytic activity and to what extent, different Hgdh variants containing single mutations were generated, expressed, and the activity of the corresponding lysates toward 2-oxoadipate (Figures 6A and S5A) and 2-oxoglutarate (Figures 6B and S5A) was determined.

All of the single variants tested were found to significantly improve Hgdh activity (*p* < 0.05 in a Welch *t*-test) (Figures 6A and S5A). Notably, A206V was the most effective mutation, increasing the activity of Hgdh toward 2-oxoadipate by 4.8-fold and, therefore, it was largely responsible for the 6.6-fold higher activity of Ep1 compared to wild-type Hgdh. Mutations A214V, A214D, E304K, and K307I improved the activity between 2.3- and 2.8-fold. It is important to note that the improvements observed in single mutants were small in comparison to those obtained in the best variants from the combinatorial library, which ranged between 73- and 126-fold. This result proves that the increments characterizing the variants obtained in the combinatorial library were largely due to a synergistic effect.

It is interesting to note that all of the variants with single mutations (except A2T, whose activity was akin to that of wild-type Hgdh) showed reduced activity (37–54% less) toward 2-oxoglutarate, the natural substrate of Hgdh (Figures 6B and S5A). Notably, V11M showed 71% less activity compared to wild-type Hgdh. These were promising results, as the study aimed to obtain a highly efficient enzyme-specific for 2-oxoadipate reduction.

### Characterization of the Best Variants

The variants Co1 (A2T/V11K/A206V/A214V/E304K), Co2 (A2T/V11K/A206V/A214D/K307I), and Co3 (A2T/V11K/A206V/A214V/V312I) from the combinatorial library (Figure 5B) were expressed in *E. coli* BL21 (DE3), purified (Figure S6), and their kinetic constants were measured using 2-oxoadipate as substrate (Table 2 and Figure S7). The *k*_cat_ values for Co1, Co2, and Co3 were improved by 151-, 124-, and 328-fold, respectively, compared to the wild-type enzyme. In contrast, the *K*_m_ values of the variants were similar to those of wild-type Hgdh in the case of Co1 and Co2, or slightly increased (3.3-fold) for Co3. Impressively, the efficiency (*k*_cat_/*K*_m_) of the best variants from the combinatorial library was improved by 2 orders of magnitude compared to the wild-type enzyme. Importantly, these results confirm that the higher activity shown by these variants is due to better catalysis of the reaction, rather than faster or stronger production of the enzyme by the screened clones.

**Table 2:** Kinetic Constants for the Reduction of 2-Oxoadipate by Wild-Type Hgdh and its Improved Variants^a^

substrate	enzyme	*k*_cat_ (s^–1^)	*K*_m_ (mM)	*k*_cat_/*K*_m_ (M^–1^ s^–1^)
2-oxoadipate	wild-type Hgdh	0.70 ± 0.03	2.96 ± 0.24	2.4 × 10^2^
	Co1	106 ± 3	4.18 ± 0.20	2.5 × 10^4^
	Co2	87 ± 3	3.05 ± 0.24	2.8 × 10^4^
	Co3	230 ± 11	9.72 ± 0.67	2.4 × 10^4^

a Reactions were carried out at 25 **°**C in 50 mM phosphate buffer, pH 8, containing 0.25 mM NADH. Results are expressed as mean ± standard deviation values of triplicate assays. *k*_cat_ and *K*_m_ are apparent values since *K*_m_ for NADH was not determined.

Interestingly, the activity of variants Co1, Co2, and Co3 (from culture lysates) toward 0.5 mM 2-oxoglutarate revealed a reduction to 0.9% (Co1), 0.6% (Co2), and 1.6% (Co3) of wild-type Hgdh activity (Figure S8). The kinetic parameters toward 2-oxoglutarate could not be determined due to the low activity observed at low substrate concentrations (data not shown). This could be due to an inhibition effect that will have to be further investigated.

It is worth mentioning that the efficiency showed by the improved Hgdh variants toward 2-oxoadipate is comparable to that shown by other (*R*)-2-hydroxyacid dehydrogenases toward their respective natural substrates, whose median *k*_cat_/*K*_m_ is 1.45 × 10^5^ M^–1^ s^–1^.^15^ However, the Hgdh variants obtained in this study revealed *K*_m_ values above the average affinity shown by (*R*)-2-hydroxyacid dehydrogenases (600 μM), suggesting weaker enzyme–substrate interactions with 2-oxoadipate.^15^

### Rationale for How the Different Mutations Improve Hgdh Activity

The current study aimed to switch the specificity of Hgdh from 2-oxoglutarate to 2-oxoadipate, which is one carbon longer than the natural substrate (Figure 2C) but exhibits analogous physicochemical properties. One can hypothesize that the catalytic pocket must be slightly enlarged to accommodate the new substrate while maintaining its characteristics, with a negatively charged patch binding the ω-carboxylate.

**Figure 7:**
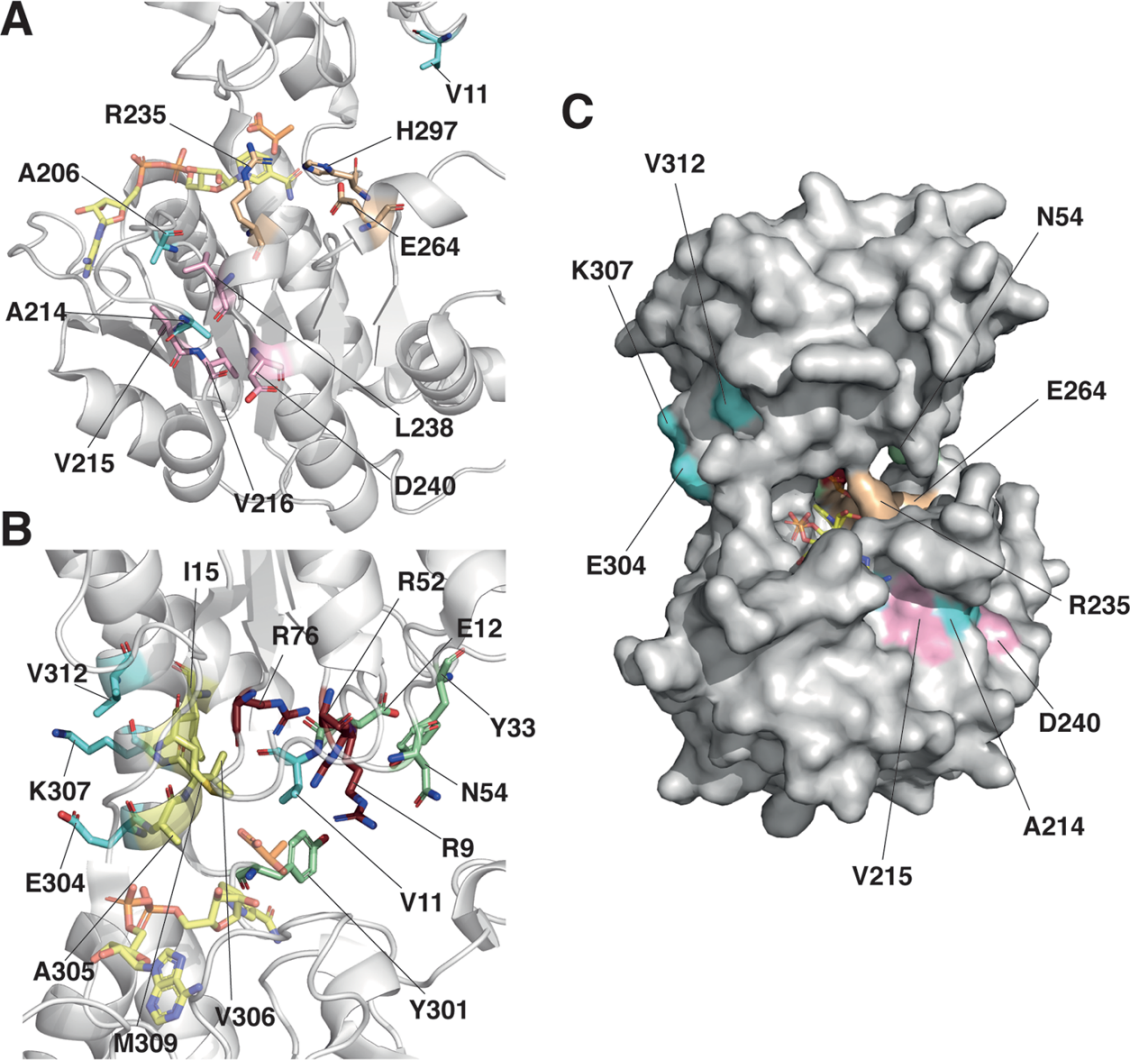
Structural basis for the improved activity in Hgdh variants. (A) Details of the structure of Hgdh (PDB ID 1XDW) showing the mutated residues V11, A206, and A214 (cyan sticks), the catalytic residues (light orange sticks), and the residues located below the catalytic arginine (pink sticks). (B) Details of the structure of Hgdh showing the mutated residues V11, E304, K307, and V312 (cyan sticks), the arginine cluster (dark red sticks), the residues forming a hydrophobic patch (yellow sticks), and the residues participating in a polar patch (green sticks). (C) Surface representation of Hgdh showing the location of the mutated residues E304, K307, V312, A214 (cyan), the catalytic residues (light orange), V215, D240 (pink), and N54 (green). In all of the panels, lactate (transparent orange sticks) and NADH (transparent yellow sticks) from the LDH structure (PDB ID 3KB6) are shown for reference.

Analysis of the Hgdh structure revealed that the beneficial mutations found in the improved variants were located mainly in two areas: (i) the loop positioned between the β-strand βD and helix 3_10_-A,^19^ and encompassing residues A206 and A214 (Figure 7A), and (ii) the helix α4^19^ (containing the mutated residues E304, K307, and V312) and residues defining the active site (Figure 7B). Even though the mechanism, by which the mutations improve the catalytic properties of Hgdh, will require further experimental data to be elucidated, the crystal structure of Hgdh (PDB ID 1XDW) was analyzed for possible cues (Figure 7).

The loop comprising residues A206 and A214 is positioned behind the area including R235 (Figure 7A), the catalytic residue that binds the α-carboxylate and the carbonyl group of the substrate, indicating its essential role in both substrate binding and activation.^19^ Residues L238 and D240 in the area containing R235 interact via hydrogen bonds with residues V215 and V216 in the loop. Therefore, it is possible that mutations A206V, A214V, and A214D affect the positioning of R235 and the surrounding structure, influencing substrate orientation and, in turn, improving catalysis.

In addition, residue A206 is buried in the structure close to the binding site of NADH (Figure 7A–C) and is part of the hydrophobic pocket that surrounds the adenine moiety of the cofactor.^19^ It is worth noting that mutation A206V implies a bulkier side chain compared to the native residue; however, the same physicochemical properties need to be maintained to improve activity toward 2-oxoadipate without compromising the role that this residue has in the structural arrangement of NADH. In comparison, residue A214 (facing the solvent) accepted a wider range of mutations, as it could be mutated to either valine or aspartate in the improved mutants.

Residues E304, K307, and V312 are located in the helix α4,^19^ facing the solvent (Figure 7B,C), whereas residues A305, V306, and M309 are located in the same α-helix, but form a hydrophobic patch together with I15, which is involved in substrate recognition. This hydrophobic patch; the arginine cluster formed by R9, R52, and R76 (binding the ω-carboxylate of the substrate); a polar patch formed by E12, Y33, and N54, as well as Y301 and F140′ (from the other monomer) delineate the catalytic pocket around the ω-carboxylate of the substrate.^19^ Therefore, it is possible that mutations E304K, K307I, and V312I can lead to subtle changes in the interactions and positioning of residues forming the hydrophobic patch, including A305, V306, and M309. These alterations can subsequently lead to better accommodation and orientation of 2-oxoadipate, ultimately, improving the activity of the enzyme toward its substrate. Furthermore, we speculate that Hgdh variants have a poorer accommodation of the natural substrate 2-oxoglutarate, leading to decreased activity. The 2-oxoglutarate is one carbon shorter than the target substrate and the correct orientation of its α-carboxylate and carbonyl group inside the catalytic pocket of Hgdh is important for the enzyme specificity.

Finally, the beneficial mutations found in this study are not located directly on the active site but on a shell behind it. The discovery of these mutations highlights the importance of the loop βD-helix-3_10_-A and helix α4 regions (following the numbering of Martins et al.^19^) as potential areas to engineer to modify interactions with the substrate and, therefore, the specificity of Hgdh and other members of the (*R*)-2-hydroxyacid dehydrogenase family.

### Concluding Remarks

The reduction of 2-oxoadipate to (*R*)-2-hydroxyadipate is a crucial reaction in metabolic pathways that produce adipic acid via α-reduction. However, existing enzymes catalyze this reaction only with low rates. In the present study, we aimed to engineer an enzyme capable of efficient 2-oxoadipate reduction, while gaining insight into the structural determinants responsible for improving enzymatic activity. During our initial assessment of the activity shown by the different candidate enzymes, we found that Pgdh was able to catalyze the reduction of 2-oxoadipate, although with a low efficiency. To the best of our knowledge, this is the first time such activity by Pgdh is reported and confirms the promiscuity of Pgdh. Following the engineering of Hgdh, three mutant variants with an outstanding ≈100-fold higher catalytic efficiency toward 2-oxoadipate were obtained. The selected approach, which combined computational analysis with random mutagenesis, generated enzymes with remarkably improved catalytic properties and proved the method’s ability to identify beneficial mutations that would be difficult to predict otherwise. Overall, the improved variants Co1, Co2, and Co3 engineered in the present study are valuable tools for the production of bio-based adipic acid. In vivo tests will be necessary to assess the actual performance of these enzymes in a microbial host endowed with the enzymatic activities of the pathway proposed in Figure 1. In this regard, an important consideration is related to the redox imbalance of the proposed pathway (i.e., 2 NAD(P)H are consumed and only one NAD(P)H is produced per mole of converted lysine). An effective evaluation of the improved Hgdh variants will rely on establishing a process able to favor NADH formation and prevent NADH shortage. One way to ensure NADH availability is ensuring high activity of the tricarboxylic acid cycle and therefore ensuring aerobic conditions and possibly respiratory metabolism. In addition, specific metabolic engineering strategies could be considered aiming at increasing the levels of NADH and the ratio NADH/NAD+, as reported by Berríos-Rivera et al.^25,26^ Likewise, specific process conditions could be implemented to specifically tune the microbial metabolism leading to increases NADH levels.^27^ In addition, we believe that the three engineered variants identified in the present study are an excellent starting point for further engineering. Moreover, all beneficial mutations described here will improve our understanding of (*R*)-2-hydroxyacid dehydrogenases.

## Methods

### Cloning of Pgdh and Hgdh

The gene sequences encoding Pgdh and Hgdh were retrieved from the National Center for Biotechnology Information database (gene ID: 945258) and the European Nucleotide Archive (accession number: ADB47349.1), respectively. The sequences were codon-optimized for expression in *E. coli.*(28) Pgdh and Hgdh genes were synthesized by GeneScript (Piscataway, NJ) and cloned in vector pET-28b, introducing a thrombin site and a histidine tag at the C-terminus. To facilitate cloning into the vector, two nucleotides had to be introduced at the beginning of the gene after the starting methionine. As a result, the gene sequence encoded an extra alanine at position 2. The pET-28b plasmids containing the genes were transformed into *E. coli* DH5α for plasmid propagation and into *E. coli* BL21 (DE3) for protein expression.

### Heterologous Expression and Purification

Pgdh, Hgdh, and Hgdh variants were expressed in *E. coli* BL21 (DE3) after transformation with the corresponding plasmids. The cells were grown in autoinduction medium (Terrific Broth base including trace elements; Formedium Ltd., Hunstanton, U.K.) supplemented with 40 μg mL^–1^ neomycin at 30 °C (for Pgdh) or 37 °C (for Hgdh) and 200 rpm for 16 h. The cells were solubilized in 50 mM Tris-HCl (pH 7.4 for Pgdh and pH 8.0 for Hgdh) containing 300 mM NaCl, 0.5 mg mL^–1^ lysozyme, 10 U mL^–1^ DNase, 0.5 mM dithiothreitol (DTT), and 10 mM imidazole. They were then sonicated (Branson 250 Digital Sonifier; Branson Ultrasonics, Brookfield, CT) and centrifuged for 20 min at 13 500 rpm. The expressed proteins were purified by affinity chromatography using a 1 mL HisTrap column (GE Healthcare, Uppsala, Sweden) and a 20–500 mM imidazole gradient in 50 mM phosphate buffer, pH 8.0, containing 300 mM NaCl and 0.5 mM DTT.

The purity of the enzymes was determined by SDS-PAGE (Figures S1 and S4) and their concentration was determined by measuring absorbance at 280 nm. The extinction coefficient (ε280) and molecular weights (*M*) used to calculate the protein concentration were retrieved from the web-based tool ProtParam (https://web.expasy.org/protparam/). They corresponded to ε280 = 41720 M^–1^ cm^–1^ and *M* = 75 kDa for dimeric Hgdh, and ε280 = 75 640 M^–1^ cm^–1^ and *M* = 176 kDa for tetrameric Pgdh. Purified enzymes were stored in 20 mM phosphate buffer (pH 7.4 for Pgdh and pH 8.0 for Hgdh) containing 10% glycerol at −80 °C.

### Determination of Kinetic Constants

The reduction of 2-oxoadipate and 2-oxoglutarate was measured by monitoring NADH oxidation (ε340 = 6220 M^–1^ cm^–1^) using a SPECTROstar Nano microplate reader (BMG Labtech, Ortenberg, Germany). All of the data obtained from the microplate reader were automatically corrected by the plate reader software for a 1 cm optical pathway. Hgdh activity was measured at room temperature in a reaction mixture containing 50 mM phosphate buffer pH 8.0, 0.25 mM NADH, and different concentrations of 2-oxoadipate and 2-oxoglutarate (from 0 to 5 mM). Pgdh activity was measured at 37 °C in the same reaction buffer as Hgdh but at pH 7.4 and containing 1 mM DTT. All enzymatic activities were measured as initial velocities from linear increments and three independent repeats were assessed. Values and standard errors for the apparent affinity constant (*K*_m_) and enzyme turnover (*k*_cat_) were obtained by nonlinear least-squares fitting of the experimental measurements to the Michaelis–Menten model using Origin software (OriginLab Corporation, Northampton, MA).

### Computational Studies

#### FuncLib Screening

Three mutational screenings were performed using the FuncLib web server (http://funclib.weizmann.ac.il/), according to the protocol described by Khersonky et al.^20^ and a monomer from the X-ray structure of Hgdh (PDB ID 1XDW, chain A).^19^ In each FuncLib calculation, we included as essential residues (i.e., the residues kept in their wild-type conformation during the calculation) the catalytic triad and the residues thought to bind the NADH cofactor (103, 106, 107, 152–177, 206, 207, 212, 233–235, 259, 264, and 297). Amino acid positions to be diversified during the FuncLib calculations included residues in the first shell around the active site (R9, R52, R76, T77, A78, G79, D81, Y101, and S300) previously proposed to be involved in substrate recognition, and those in the second shell (V11, E12, I15, F16, R100, P296, L298, G299, Y301, and M309). The residues selected for each FuncLib calculation were: (i) scan1: R52, T77–G79, D81, Y101, and S300; (ii) scan2: R9, E12, R52, R76–G79, D81, R100, Y101, P296, L298–S300, and M309; and (iii) scan3: R9, V11, I15, F16, R52, T77, R100, L298, S300, Y301, and M309. Default parameters were used to run the calculations,^20^ and an in-house Python script was employed to plot the percentage of mutations at each selected amino acid position of multipoint mutants.

#### In Silico Saturation Mutagenesis

We employed the FoldX energy function^21^ to perform in silico saturation mutagenesis using MutateX, an automated pipeline that we recently developed.^22^ Thus, we employed the same protocol we had applied to other proteins^29,30^ to estimate the changes caused by the selected mutations on the structural stability of Hgdh. To this end, we performed the calculations on a monomer derived from the X-ray structure of Hgdh (PDB ID 1XDW, monomer in chain A). MutateX calculated changes in the free energy of folding as average ΔΔ*GS*, i.e., differences in Δ*G* between the mutant and the wild-type variant, for each possible mutation at each amino acid position, over five independent runs.

#### Saturation Mutagenesis

Saturation mutagenesis was carried out on residues R9, V11, R52, T77, A78, D81, Y101, and S300. To construct each library, a mutagenic PCR was made using the pET28b-Hgdh plasmid as template and four specific primers (three forward mutagenic primers and one reverse primer, 3). The forward primers were designed to incorporate the degenerate codons NDT (N = A/T/C/G, D = no C) and VHG (V = no T, H = no G), and the TGG codon at selected amino acid positions.^28^ PCR reactions were carried out in a final volume of 20 μL containing 0.2 mM dNTPs, 0.02 U μL^–1^ Phusion HF DNA polymerase, 2.5 ng μL^–1^ template, 0.2 μM reverse primer, and 0.2 μM of the three forward mutagenic primers mixed at a previously reported ratio.^31^ Reaction conditions were as follows: (i) a hot start of 98 °C for 2 min; (ii) 24 cycles at 98 °C for 10 s, 62 °C for 1 min, and 72 °C for 1 min 45 s; and (iii) a final cycle at 72 °C for 10 min. The generated plasmids were digested with *Dpn*I (FastDigest *Dpn*I, Thermo Scientific, Waltham, MA) at 37 °C for 20 min, dialyzed against water using an MF-Millipore Membrane Filter (0.025 μm pore size; Merck Millipore, Billerica, MA), and transformed into *E. coli*. The plasmids from ten colonies of each library were sequenced to evaluate the genetic variability at the targeted residues. Around 90 colonies per library were randomly selected, grown, and screened.

### Generation of the V11M/V11K Mutant Library by ep-PCR

Whole-gene ep-PCR was performed using the GeneMorph II Random Mutagenesis kit (Agilent Technologies, La Jolla, CA), an equal mix of pET28b-Hgdh-V11K and pET28b-Hgdh-V11M plasmids as template, and the primers ep_PCR_F and ep_PCR_R (Table S3). The PCR conditions were set according to the manufacturer’s instructions to obtain 2–3 mutations/kb. The vector pET-28b (+) was amplified and linearized in a PCR mixture containing 0.2 mM dNTPs, 0.02 U μL^–1^ Phusion HF DNA polymerase, 0.2 ng μL^–1^ template, and 0.5 μM primers Vector_F and Vector_R (Table S3). Temperatures and times were according to the manufacturer’s recommendations. The linearized pET-28b (+) vector and the pool of mutated *hgdh* products from the ep-PCR were digested with *Dpn*I, assembled in a Gibson reaction (using a vector:fragment ratio of 1:5^32^) and transformed in *E. coli* BL21 (DE3). Around 2000 colonies obtained from the transformation were randomly selected, grown, and screened.

### Generation of the Combinatorial Library

To generate the combinatorial library, two independent PCRs were carried out: a first PCR amplified the *hgdh* gene fragment encoding residues 1 to 206 using primers A206V_D and epPCR_R (Table S3), while a second PCR amplified the *hgdh* gene fragment encoding residues 206 to 346 using primers A206V_R and epPCR_D_A2T (Table S3). A mix of plasmids obtained from the library generated by ep-PCR and including potentially beneficial mutations was used as template (Table S2). The Phusion High-Fidelity DNA polymerase was used, and the PCR conditions were set according to the manufacturer’s instructions. The PCR products were then digested with *Dpn*I and assembled in a Gibson reaction^32^ together with the linearized vector pET-28b. The Gibson assembly reaction used a vector:fragment 1:fragment 2 ratio of 1:3:3. The resulting plasmids were transformed in *E. coli* BL21 (DE3). Around 250 colonies obtained from the transformation were randomly selected, grown, and screened as previously explained.

### Microtiter Plate-Based Growth and High-Throughput Screening of Libraries

The activity assay based on NADH oxidation used for determining the kinetic constants of Pgdh and Hgdh was adapted for use in high-throughput screening with culture lysates. The possible background activity observed using lysates from *E. coli* BL21 (DE3) pET28b and *E. coli* BL21 (DE3) pET28b_bsa (expressing bovine serum albumin) cultures, as well as that from *E. coli* BL21 (DE3) pET28b-Hgdh cultures taken at 0 h of induction was recorded and monitored. All three reactions showed a negligible and stable background activity. To assess the suitability of the assay, the coefficient of variation was determined using the activity data of lysates from *E. coli* BL21 (DE3)-Hgdh cells grown and induced in a 96-well plate (Figure S4).

Colonies from the different libraries were randomly selected and inoculated into 96 deep-well plates containing 0.5 mL LB medium supplemented with 40 μg mL^–1^ neomycin per well. After incubation at 37 °C and 200 rpm for 18 h, an aliquot of 30 μL per well was transferred into a new 96 deep-well plate containing 0.5 mL of autoinduction medium supplemented with 40 μg mL^–1^ neomycin per well. The cultures were further incubated at 37 °C and 200 rpm for 18 h. After centrifuging the cultures, the pellets were frozen at −20 °C for 24 h. The thawed pellets were then resuspended in lysis buffer (50 mM phosphate buffer pH 8.0, 300 mM NaCl, 5% glycerol, 0.5 mg mL^–1^ lysozyme, and 10 U mL^–1^ DNAse), incubated at room temperature for 1 h, and centrifuged (2000*g* for 10 min). The activity of the lysates toward 2-oxoadipate (or 2-oxoglutarate) was measured by mixing an appropriate aliquot of lysate with the reaction buffer (50 mM phosphate buffer pH 8.0, 0.25 mM NADH, 0.5 mM or 2 mM 2-oxoadipate or 2-oxoglutarate) in an ultraviolet (UV)-transparent 96-well plate (Greiner Bio-One International AG, Kremsmünster, Austria) and measuring the decrease in absorbance at 340 nm using a SPECTROstar Nano microplate reader. Activity was determined as the linear slope generated by NADH oxidation (ε340 = 6220 M^–1^ cm^–1^). Six cultures of the parent strain were assayed per 96-well plate and used as reference. Variants showing higher activity than the parent enzyme (average activity plus two standard deviations) were rescreened. To this end, aliquots from each LB culture containing the desired variants were streaked on an LB agar plate supplemented with 40 μg mL^–1^ neomycin. Then, four colonies per variant were independently screened as described above. A *t*-test was used to determine the significance of the obtained data using Origin software (OriginLab Corporation, Northampton, MA).

### Design and Production of Single Mutants by Site-Directed Mutagenesis

Hgdh variants containing single mutations (A2T, A206V, A214D, A214V, E304K, K307I, and V312I) were produced by PCR using the pET28b-Hgdh plasmid as template, and primers designed to complement the DNA region containing the desired mutation (Table S3). PCR mixtures contained 0.4 ng μL^–1^ template DNA, 250 μM of each dNTP, 125 ng of both direct and reverse primers, 1 unit of Phusion High-Fidelity DNA polymerase (Thermo Scientific), and the manufacturer’s reaction buffer. Reaction conditions were as follows: (i) a hot start at 98 °C for 1 min; (ii) 18 cycles at 98 °C for 20 s, 58 °C for 50 s, and 72 °C for 6 min; and (iii) a final cycle at 72 °C for 10 min. The resulting plasmids were digested with *Dpn*I, dialyzed against water using an MF-Millipore Membrane Filter (0.025 μm pore size), and transformed into *E. coli* DH5α. The mutated plasmid from a positive clone of each variant was sequenced (Macrogen, Amsterdam, The Netherlands) to confirm that the desired mutations had been properly introduced. The verified plasmids were then transformed into *E. coli* BL21 (DE3) for protein expression.

## Data Availability

The scripts, inputs, and outputs of the computational analysis included in this study are available in the GitHub repository associated with the publication https://github.com/ELELAB/hgdh_engineering.

## Supporting Information Available

SDS-PAGE results of the purified Pgdh (A) and Hgdh (B) (Figure S1); kinetics of Hgdh and Pgdh with 2-oxoadipate and 2-oxoglutarate (Figure S2); barplots showing the percentage of mutant variants from the first and second FuncLib calculation (Figure S3); coefficient of variation of the high-throughput screening activity assay used in protein engineering experiments (Figure S4); percentage of functional variants found in the libraries generated by saturation mutagenesis (Table S1); plasmids used as templates for the generation of the combinatorial library and the mutations they contained (Table S2); primers used in this study (Table S3); activity of single mutant variants (Figure S5); SDS-PAGE results of the purified Hgdh variants Co1, Co2, and Co3 (Figure S6); kinetics of the improved variants with 2-oxoadipate (Figure S7); and activity of the improved variants Co1, Co2, and Co3 toward 2- oxoglutarate (Figure S8) (PDF)

## References

[ref1] Polen T.; Spelberg M.; Bott M. Toward biotechnological production of adipic acid and precursors from biorenewables. J. Biotechnol. 2013, 167, 75–84. 10.1016/j.jbiotec.2012.07.008. 22824738

[ref2] Deng Y.; Ma L.; Mao Y. Biological production of adipic acid from renewable substrates: Current and future methods. Biochem. Eng. J. 2016, 105, 16–26. 10.1016/j.bej.2015.08.015.

[ref3] Alini S.; Basile F.; Blasioli S.; Rinaldi C.; Vaccari A. Development of new catalysts for N2O-decomposition from adipic acid plant. Appl. Catal., B 2007, 70, 323–329. 10.1016/j.apcatb.2005.12.031.

[ref4] Suitor J. T.; Varzandeh S.; Wallace S. One-Pot Synthesis of Adipic Acid from Guaiacol in *Escherichia coli*. ACS Synth. Biol. 2020, 9, 2472–2476. 10.1021/acssynbio.0c00254. 32786923

[ref5] Kruyer N. S.; Peralta-Yahya P. Metabolic engineering strategies to bio-adipic acid production. Curr. Opin. Biotechnol. 2017, 45, 136–143. 10.1016/j.copbio.2017.03.006. 28365404

[ref6] Skoog E.; Shin J. H.; Saez-Jimenez V.; Mapelli V.; Olsson L. Biobased adipic acid - The challenge of developing the production host. Biotechnol. Adv. 2018, 36, 2248–2263. 10.1016/j.biotechadv.2018.10.012. 30389426

[ref7] Karlsson E.; Shin J. H.; Westman G.; Eriksson L. A.; Olsson L.; Mapelli V. In silico and in vitro studies of the reduction of unsaturated α,β bonds of trans-2-hexenedioic acid and 6-amino-trans-2-hexenoic acid – Important steps towards biobased production of adipic acid. PLoS One 2018, 13, e0193503 10.1371/journal.pone.0193503. 29474495 PMC5825115

[ref8] Burgard A.; Pharkya P.; Osterhout R. E. Microoganisms for the Production of Adipic Acid and Other Compounds. U.S. Patent, US7,799,5452016.

[ref9] Brautaset T.; Ellingsen T. E. Lysine, Industrial Uses and Production. In Comprehensive Biotechnology, Moo-Young M., Ed.; Elsevier: 2011; pp 541–554.

[ref10] Parthasarathy A.; Pierik A. J.; Kahnt J.; Zelder O.; Buckel W. Substrate specificity of 2-hydroxyglutaryl-CoA dehydratase from Clostridium symbiosum: toward a bio-based production of adipic acid. Biochemistry 2011, 50, 3540–3550. 10.1021/bi1020056. 21434666

[ref11] Reitman Z. J.; Choi B. D.; Spasojevic I.; Bigner D. D.; Sampson J. H.; Yan H. Enzyme redesign guided by cancer-derived IDH1 mutations. Nat. Chem. Biol. 2012, 8, 887–889. 10.1038/nchembio.1065. 23001033 PMC3487689

[ref12] Baynes B. M.; Geremia J. M. L.; Lippow S. M. Biological Synthesis of 6-Aminocaproic Acid from Carbohydrate Feedstocks. U.S. Patent, US8,404,4652015.

[ref13] Zhang Y.; Ashok S.; Seol E.; Ainala S. K.; Lee S.-G.; Madan B.; Xu J.-H.; Park S. NADH-dependent lactate dehydrogenase from *Alcaligenes eutrophus* H16 reduces 2-oxoadipate to 2-hydroxyadipate. Biotechnol. Bioprocess Eng. 2014, 19, 1048–1057. 10.1007/s12257-014-0381-1.

[ref14] Parthasarathy A. Substrates and Mechanism of 2-Hydroxyglutaryl-CoA-Dehydratase from Clostridium Symbiosum; Universität Marburg, 2009.

[ref15] Matelska D.; Shabalin I. G.; Jablonska J.; Domagalski M. J.; Kutner J.; Ginalski K.; Minor W. Classification, substrate specificity and structural features of D-2-hydroxyacid dehydrogenases: 2HADH knowledgebase. BMC Evol. Biol. 2018, 18, 199 10.1186/s12862-018-1309-8. 30577795 PMC6303947

[ref16] Antonyuk S. V.; Strange R. W.; Ellis M. J.; Bessho Y.; Kuramitsu S.; Inoue Y.; Yokoyama S.; Hasnain S. S. Structure of D-lactate dehydrogenase from *Aquifex aeolicus* complexed with NAD(+) and lactic acid (or pyruvate). Acta Crystallogr., Sect. F: Struct. Biol. Cryst. Commun. 2009, 65, 1209–1213. 10.1107/S1744309109044935. PMC2802865 20054113

[ref17] Stoll V. S.; Kimber M. S.; Pai E. F. Insights into substrate binding by D-2-ketoacid dehydrogenases from the structure of Lactobacillus pentosus D-lactate dehydrogenase. Structure 1996, 4, 437–447. 10.1016/S0969-2126(96)00049-4. 8740366

[ref18] Dengler U.; Niefind K.; Kieβ M.; Schomburg D. Crystal Structure of a Ternary Complex of D-2-Hydroxyisocaproate Dehydrogenase from Lactobacillus casei, NAD (+)‡ and 2-oxoisocaproate at 1.9 Å Resolution. J. Mol. Biol. 1997, 267, 640–660. 10.1006/jmbi.1996.0864. 9126843

[ref19] Martins B. M.; Macedo-Ribeiro S.; Bresser J.; Buckel W.; Messerschmidt A. Structural basis for stereo-specific catalysis in NAD(+)-dependent (R)-2-hydroxyglutarate dehydrogenase from *Acidaminococcus fermentans*. FEBS J. 2004, 272, 269–281. 10.1111/j.1432-1033.2004.04417.x. 15634349

[ref20] Khersonsky O.; Lipsh R.; Avizemer Z.; Ashani Y.; Goldsmith M.; Leader H.; Dym O.; Rogotner S.; Trudeau D. L.; Prilusky J.; Amengual-Rigo P.; Guallar V.; Tawfik D. S.; Fleishman S. J. Automated Design of Efficient and Functionally Diverse Enzyme Repertoires. Mol. Cell 2018, 72, 178–186.e5. 10.1016/j.molcel.2018.08.033. 30270109 PMC6193528

[ref21] Schymkowitz J.; Borg J.; Stricher F.; Nys R.; Rousseau F.; Serrano L. The FoldX web server: an online force field. Nucleic Acids Res. 2005, 33, W382–W388. 10.1093/nar/gki387. 15980494 PMC1160148

[ref22] Tiberti M.; Terkelsen T.; Degn K.; Beltrame L.; Cremers T. C.; da Piedade I.; Di Marco M.; Maiani E.; Papaleo E. MutateX: an automated pipeline for *in silico* saturation mutagenesis of protein structures and structural ensembles. Brief Bioinform. 2022, 23, bbac074 10.1093/bib/bbac074. 35323860

[ref23] Zhao G.; Winkler M. E. A Novel a-Ketoglutarate Reductase Activity of the serA-Encoded 3-Phosphoglycerate Dehydrogenase of *Escherichia coli* K-12 and Its Possible Implications for Human 2-Hydroxyglutaric Aciduria. J. Bacteriol. 1996, 178, 232–239. 10.1128/jb.178.1.232-239.1996. 8550422 PMC177644

[ref24] Grant G. A. Contrasting catalytic and allosteric mechanisms for phosphoglycerate dehydrogenases. Arch. Biochem. Biophys. 2012, 519, 175–185. 10.1016/j.abb.2011.10.005. 22023909 PMC3294004

[ref25] Berríos-Rivera S. J.; Bennett G. N.; San K.-Y. Metabolic engineering of *Escherichia coli*: increase of NADH availability by overexpressing an NAD(+)-dependent formate dehydrogenase. Metab. Eng. 2002, 4, 217–229. 10.1006/mben.2002.0227. 12616691

[ref26] Berríos-Rivera S. J.; Bennett G. N.; San K.-Y. The Effect of Increasing NADH Availability on the Redistribution of Metabolic Fluxes in *Escherichia coli* Chemostat Cultures. Metab. Eng. 2002, 4, 230–237. 10.1006/mben.2002.0228. 12616692

[ref27] Schuhmacher T.; Löffler M.; Hurler T.; Takors R. Phosphate limited fed-batch processes: Impact on carbon usage and energy metabolism in *Escherichia coli*. J. Biotechnol. 2014, 190, 96–104. 10.1016/j.jbiotec.2014.04.025. 24833421

[ref28] Puigbo P.; Guzman E.; Romeu A.; Garcia-Vallve S. OPTIMIZER: a web server for optimizing the codon usage of DNA sequences. Nucleic Acids Res. 2007, 35, W126–W131. 10.1093/nar/gkm219. 17439967 PMC1933141

[ref29] Saez-Jimenez V.; Marsic Z. S.; Lambrughi M.; Shin J. H.; van Havere R.; Papaleo E.; Olsson L.; Mapelli V. Structure-function investigation of 3-methylaspartate ammonia lyase reveals substrate molecular determinants for the deamination reaction. PLoS One 2020, 15, e0233467 10.1371/journal.pone.0233467. 32437404 PMC7241714

[ref30] Fas B. A.; Maiani E.; Sora V.; Kumar M.; Mashkoor M.; Lambrughi M.; Tiberti M.; Papaleo E. The conformational and mutational landscape of the ubiquitin-like marker for autophagosome formation in cancer. Autophagy 2020, 17, 2818–2841. 10.1080/15548627.2020.1847443. 33302793 PMC8525936

[ref31] Kille S.; Acevedo-Rocha C. G.; Parra L. P.; Zhang Z. G.; Opperman D. J.; Reetz M. T.; Acevedo J. P. Reducing codon redundancy and screening effort of combinatorial protein libraries created by saturation mutagenesis. ACS Synth. Biol. 2013, 2, 83–92. 10.1021/sb300037w. 23656371

[ref32] Gibson D. G.; Young L.; Chuang R. Y.; Venter J. C.; Hutchison C. A. III; Smith H. O. Enzymatic assembly of DNA molecules up to several hundred kilobases. Nat Methods 2009, 6, 343–345. 10.1038/nmeth.1318. 19363495

