# Supplementary material for: Directed Evolution of (R)-2-Hydroxyglutarate Dehydrogenase Improves 2-Oxoadipate Reduction by 2 Orders of Magnitude
Source: ACS Synth Biol. 2022 Aug 8;11(8):2779–90. doi: 10.1021/acssynbio.2c00162 (PMC9396657; doi:10.1021/acssynbio.2c00162)
Supplement: Supplementary file 1 — sb2c00162_si_001.pdf [file sb2c00162_si_001.pdf]

Supplementary Information

Directed evolution of (*R*)-2-hydroxyglutarate dehydrogenase improves 2-oxoadipate reduction by two order of magnitude

Authors: Veronica Saez-Jimenez[1], Simone Scrima[2,3], Matteo Lambrughi[2], Elena Papaleo[2,3], Valeria Mapelli[1], Martin K. M. Engqvist[4], Lisbeth Olsson[1]*

Affiliations:

[1]Division of Industrial Biotechnology, Department of Biology and Biological Engineering, Chalmers University of Technology, Gothenburg, Sweden

[2]Cancer Structural Biology, Danish Cancer Society Research Center, Copenhagen, Denmark

[3]Cancer Systems Biology, Section for Bioinformatics, Department of Health and Technology, Technical University of Denmark, Lyngby, Denmark

[4]Division of Systems and Synthetic Biology, Department of Biology and Biological Engineering, Chalmers University of Technology, Gothenburg, Sweden

*Corresponding author:

E-mail: lisbeth.olsson@chalmers.se (LO)

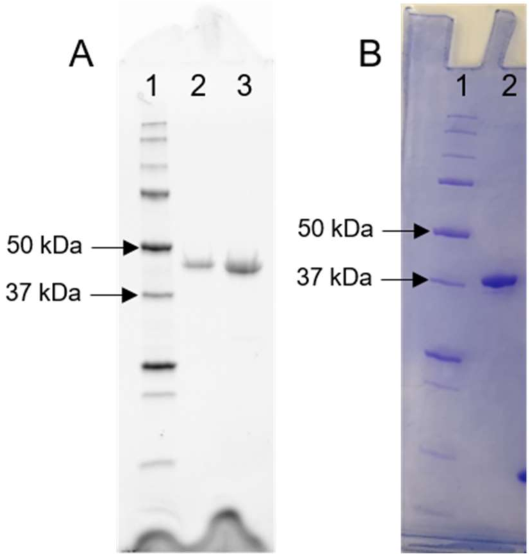

Figure S1. SDS-PAGE results of the purified Pgdh (A) and Hgdh (B). A, lane 1 shows the protein marker and lane 2 and 3 show 2 µg and 4 µg of Pgdh (44 kDa), respectively. Proteins in the gel were visualized using rapid fluorescent detection (TGX Stain-Free gels and GelDoc Imaging System, BioRad). B, lane 1 shows the protein marker and lane 2 shows 2 µg of Hgdh (37 kDa). Proteins in the gel were visualized using Coomassie Brilliant Blue staining method.

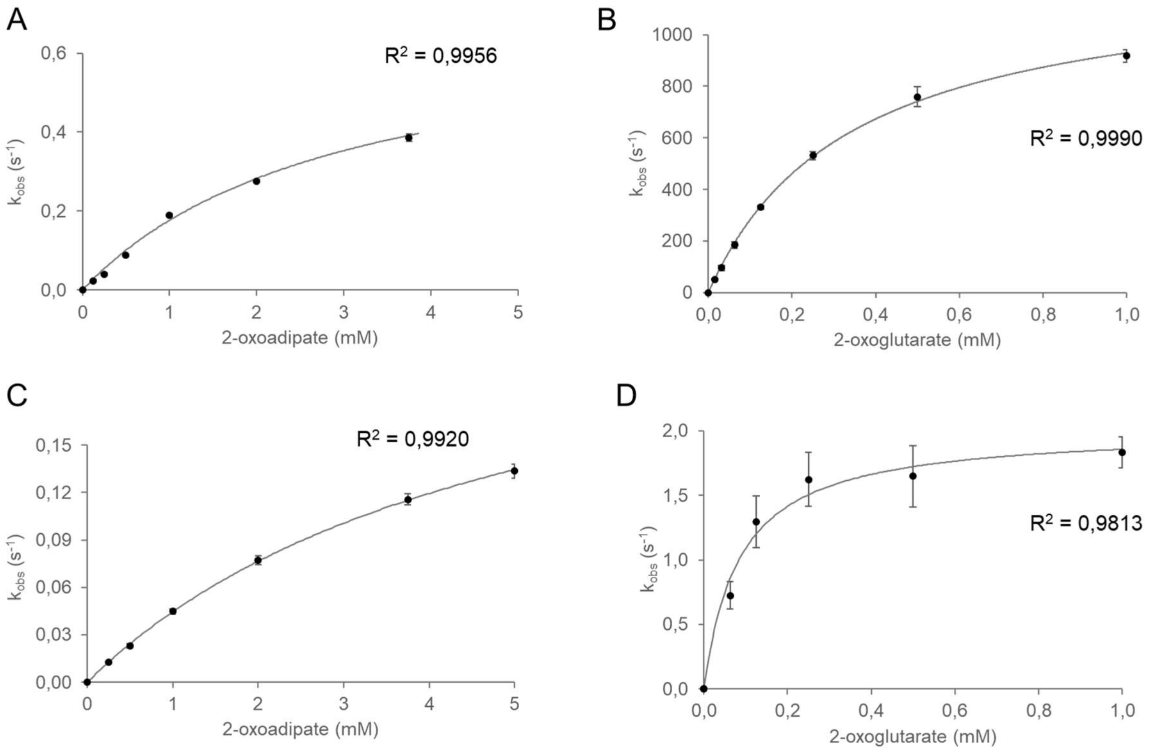

Figure S2. Kinetics of Hgdh and Pgdh with 2-oxoadipate and 2-oxoglutarate. A and B, kinetics of Hgdh with 2-oxoadipate and 2-oxoglutarate, respectively. C and D, kinetics of Pgdh with 2-oxoadipate and 2-oxoglutarate, respectively. Reactions with Hgdh were carried out at 25°C in 50 mM phosphate buffer, pH 8, containing 0.25 mM NADH. Reactions with Pgdh were carried out at 37°C in 50 mM phosphate buffer, pH 7.4, containing 0.25 mM NADH and 1 mM DTT. Results are means ± standard deviation values of triplicate assays.

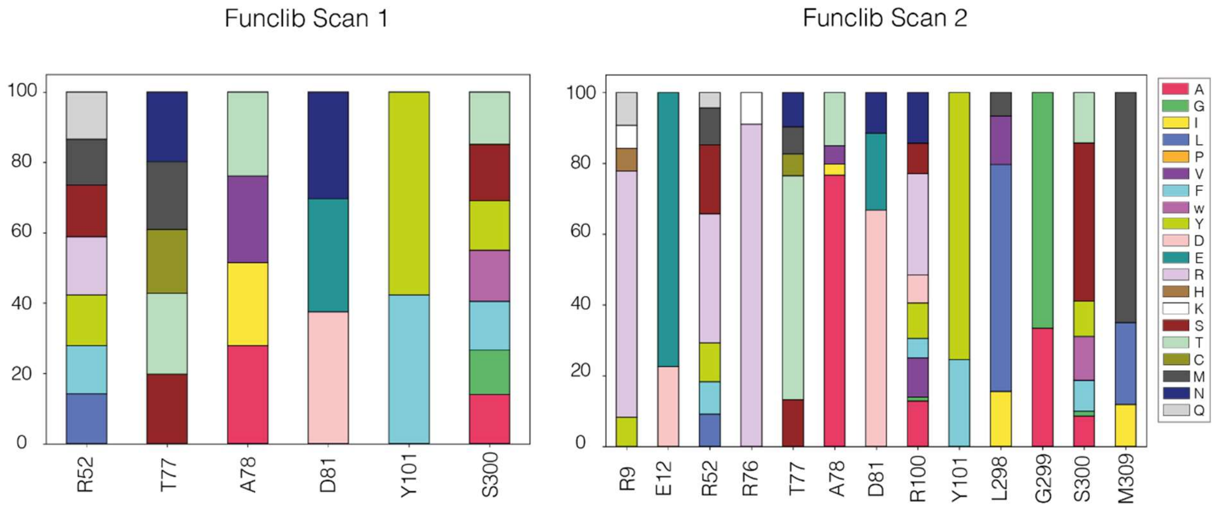

Figure S3. Barplots showing the percentage of mutant variants from the first and second FuncLib calculation. The bar plots show the percentage of mutant variants at each amino acid position selected to be diversified in the first and second FuncLib calculation.

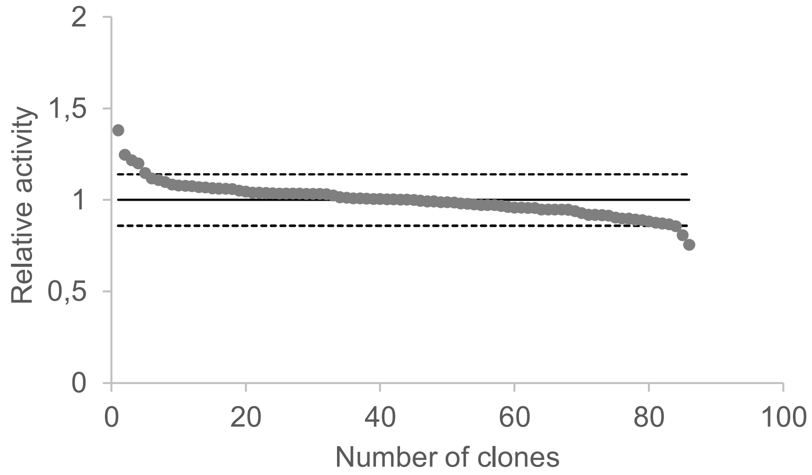

Figure S4. Coefficient of variation of the high through-put screening activity assay used in protein engineering experiments. Activities of the wild-type Hgdh plotted in descending order. *E. coli* BL21 (DE3) cells were transformed with pET28b-Hgdh and plated. Individual colonies were picked, inoculated, and grown in autoinduction media in a 96 deep-well plate. The activity of the corresponding lysates towards 2-oxoadipate was measured as the decrease of absorbance at 340 nm due to NADH oxidation (ε340 = 6220 M^{-1} cm^{-1}). Dashed lines indicate the coefficient of variance of the assay (14.1 %).

Table S1. Percentage of functional variants found in the libraries generated by saturation mutagenesis.

Library	% Functional variants[a]
R9	10
V11	30
R52	2
T77	4
D81	16
Y101	14
S300	8

[a] Functional variants were defined as the ones showing an activity higher than the average activity of the wild-type Hgdh minus two standard deviations.

Table S2. Plasmids used as templates for the generation of the combinatorial library and the mutations they contained.

**Templates used for the amplification of the region encoding residues 1 to 206**						
**Clone**	**Activity***	**Mutations included**				
Ep4	2.3		V11K	A18V	V152I	K307I
Ep8	1.9	A2T	V11M	K131T		
Ep9	1.8		V11K	T121I		
Ep10	1.8		V11K	C19Y	D32N	
Ep11	1.8		V11K	F138I		
Ep12	1.5		V11K	T148N	A206T	
Ep15	1.3		V11K	R156H		
Parent V11K			V11K			
Parent V11M			V11M			
**Templates used for the amplification of the region encoding residues 206 to 346**						
**Clone**	**Activity***	**Mutations included**				
Ep1	4.9	A2T	V11K	A206V		
Ep2	3.1		V11K	A206V	R218C	
Ep3	2.8		V11K	A214V	V312I	
Ep4	2.3		V11K	A18V	V152I	K307I
Ep5	2.2		V11K	A214D		
Ep6	2.2		V11M		E304K	
Parent V11K			V11K			

Plasmids were isolated from clones obtained in the library generated by error-prone PCR that showed improved activity compared with the parents Hgdh V11M and Hgdh V11K.
*The average of the activity found for the parents V11M and V11K was normalized to 1 and used as reference to calculate the improvement in the activity observed in the clones.

Table S3. Primers used in this study.

Primer name	Primer sequence (5' to 3')*	Experiment
9_F_NDT	GCTAAAGTTCTGTGTTATGGTGTA**NDT**GATGTAGAACTGCCGATTTTTG	Saturation mutagenesis of Arg9
9_F_VHG	GCTAAAGTTCTGTGTTATGGTGTA**VHG**GATGTAGAACTGCCGATTTTTG	
9_F_TGG	GCTAAAGTTCTGTGTTATGGTGTA**TGG**GATGTAGAACTGCCGATTTTTG	
9_R	CACCATAACACAGAACTTTAGCCATGGTATATCTC	
11_F_NDT	CTGTGTTATGGTGTACGTGAT**NDT**GAACTGCCGATTTTTGAAGCC	Saturation mutagenesis of Val11
11_F_VHG	CTGTGTTATGGTGTACGTGAT**VHG**GAACTGCCGATTTTTGAAGCC	
11_F_TGG	CTGTGTTATGGTGTACGTGAT**TGG**GAACTGCCGATTTTTGAAGCC	
11_R	CACGTACACCATAACACAGAACTTTAGCC	
52_F_NDT	CTTTGATGCGGTTATCCTG**NDT**GGCAACTGCTTCGCC	Saturation mutagenesis of Arg52
52_F_VHG	CTTTGATGCGGTTATCCTG**VHG**GGCAACTGCTTCGCC	
52_F_TGG	CTTTGATGCGGTTATCCTG**TGG**GGCAACTGCTTCGCC	
52_R	CAGGATAACCGCATCAAAGCCAGCAGCCATTTC	
77_F_NDT	GTAAAATACATCCTGACCCGT**NDT**GCCGGCACCGATCAC	Saturation mutagenesis of Thr77
77_F_VHG	GTAAAATACATCCTGACCCGT**VHG**GCCGGCACCGATCAC	
77_F_TGG	GTAAAATACATCCTGACCCGT**TGG**GCCGGCACCGATCAC	
77_R	CGGGTCAGGATGTATTTTACGCCCAGTTTTTTGTAAATG	
81_F_NDT	CCCGTACCGCCGGCACC**NDT**CACATCGATAAAGAATATGCC	Saturation mutagenesis of Asp81
81_F_VHG	CCCGTACCGCCGGCACC**VHG**CACATCGATAAAGAATATGCC	
81_F_TGG	CCCGTACCGCCGGCACC**TGG**CACATCGATAAAGAATATGCC	
81_R	GTGCCGGCGGTACGGGTCAGGATGTATTTTAC	
101_F_NDT	CGATGGCTTTCGTTCCGCGT**NDT**TCCCCGAACGCCATTGC	Saturation mutagenesis of Tyr101
101_F_VHG	CGATGGCTTTCGTTCCGCGT**VHG**TCCCCGAACGCCATTGC	
101_F_TGG	CGATGGCTTTCGTTCCGCGT**TGG**TCCCCGAACGCCATTGC	
101_R	CGGAACGAAAGCCATCGGGAAGCCC	
300_F_NDT	CACCCCGCACCTGGGC**NDT**TACACCGACGAAGCCG	Saturation mutagenesis of Ser300
300_F_VHG	CACCCCGCACCTGGGC**VHG**TACACCGACGAAGCCG	
300_F_TGG	CACCCCGCACCTGGGC**TGG**TACACCGACGAAGCCG	
300_R	GCCCAGGTGCGGGGTGATCAGAACACGC	
epPCR_D	CCCCTCTAGAAATAATTTTGTTTAACTTTAAGAAGGAGATATACCATG	Error-prone PCR
epPCR_RC	CGGATCTCAGTGGTGGTGGTG	
Vector_D	CACCACCACCACCACCACTG	
Vector_RC	CATGGTATATCTCCTTCTTAAAGTTAAACAAAATTATTTCTAGAGGGG	
A206V_D	GACATCATCACCATCCAC**GTT**CCGTACATCAAAGAAAACG	Combinatorial library (Fragment residues 1 - 206)
epPCR_RC	CGGATCTCAGTGGTGGTGGTG	
A206V_RC	CGTTTTCTTTGATGTACGG**AAC**GTGGATGGTGATGATGTC	

epPCR_D_A2T	CCCCTCTAGAAATAATTTTGTTTAACTTTAAGAAGGAGATATACCATG**A**CT	Combinatorial library (Fragment residues 206 -346)
D_A2T	GAAGGAGATATACCATG**A**CTAAAGTTCTGTGTTATGGTGTACG	Site-directed mutagenesis for A2T
RC_A2T	CGTACACCATAACACAGAACTTTAG**T**CATGGTATATCTCCTTC	
A206V_D	GACATCATCACCATCCACG**T**TCCGTACATCAAAGAAAACG	Site-directed mutagenesis for A206V
A206V_RC	CGTTTTCTTTGATGTACGGA**A**CGTGGATGGTGATGATGTC	
D_A214D	CCGTACATCAAAGAAAACGGCG**A**TGTGGTTACCCGC	Site-directed mutagenesis for A214D
RC_A214D	GCGGGTAACCACA**T**CGCCGTTTTCTTTGATGTACGG	
D_A214V	CCGTACATCAAAGAAAACGGCG**T**TGTGGTTACCCGC	Site-directed mutagenesis for A214V
RC_A214V	GCGGGTAACCACA**A**CGCCGTTTTCTTTGATGTACGG	
D_E304K	GCTCCTACACCGAC**A**AAGCCGTAAAAAACATGGTGG	Site-directed mutagenesis for E304K
RC_E304K	CCACCATGTTTTTTACGGCTT**T**GTCGGTGTAGGAGC	
D_K307I	CCGACGAAGCCGTAA**T**AAACATGGTGGAAGTTTCC	Site-directed mutagenesis for K307I
RC_K307I	GGAAACTTCCACCATGTTT**A**TTACGGCTTCGTCGG	
D_V312I	GCCGTAAAAAACATGGTGGAA**A**TTTCCTACCAGAACCTG	Site-directed mutagenesis for V312I
RC_V312I	CAGGTTCTGGTAGGAAA**T**TTCCACCATGTTTTTTACGGC	

*The changed triplets (underlined) and the mutations introduced (bold) are indicated in the sequences.

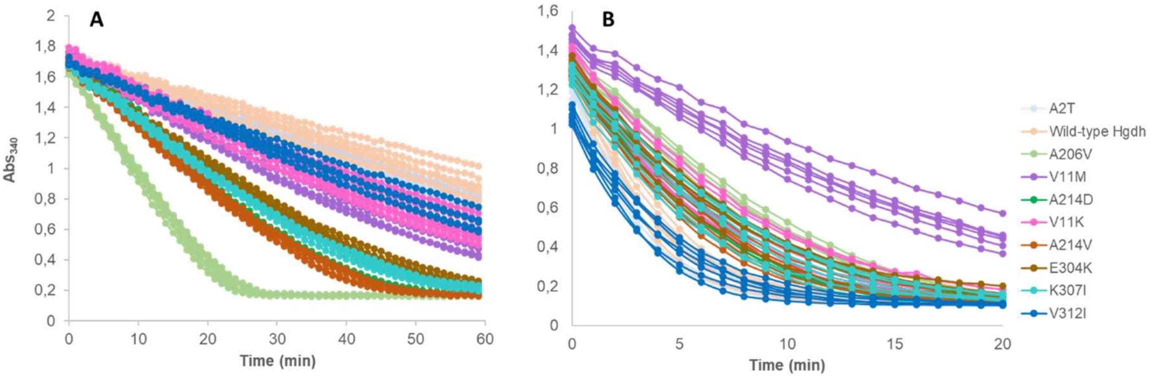

Figure S5. Activity of single mutant variants. A) Activity of the variants towards 2-oxoadipate. B) Activity of the variants towards 2-oxoglutarate. Reactions were carried out at 25°C in 50 mM phosphate buffer, pH 8, containing 0.5 mM 2-oxoadipate or 2-oxoglutarate, 0.25 mM NADH, and an appropriate aliquot of culture lysate.

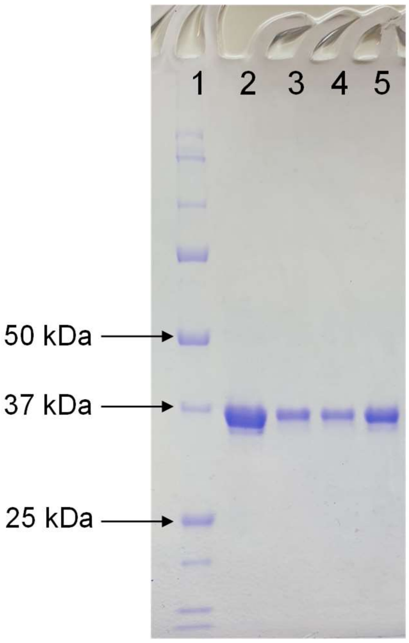

Figure S6. SDS-PAGE results of the purified Hgdh variants Co1, Co2 and Co3. Lane 1 shows the protein marker and lanes 2, 3, 4 and 5 show the wild-type Hgdh, Co1, Co2 and Co3 variants (37 kDa), respectively. Proteins in the gel were visualized using Coomassie Brilliant Blue staining method.

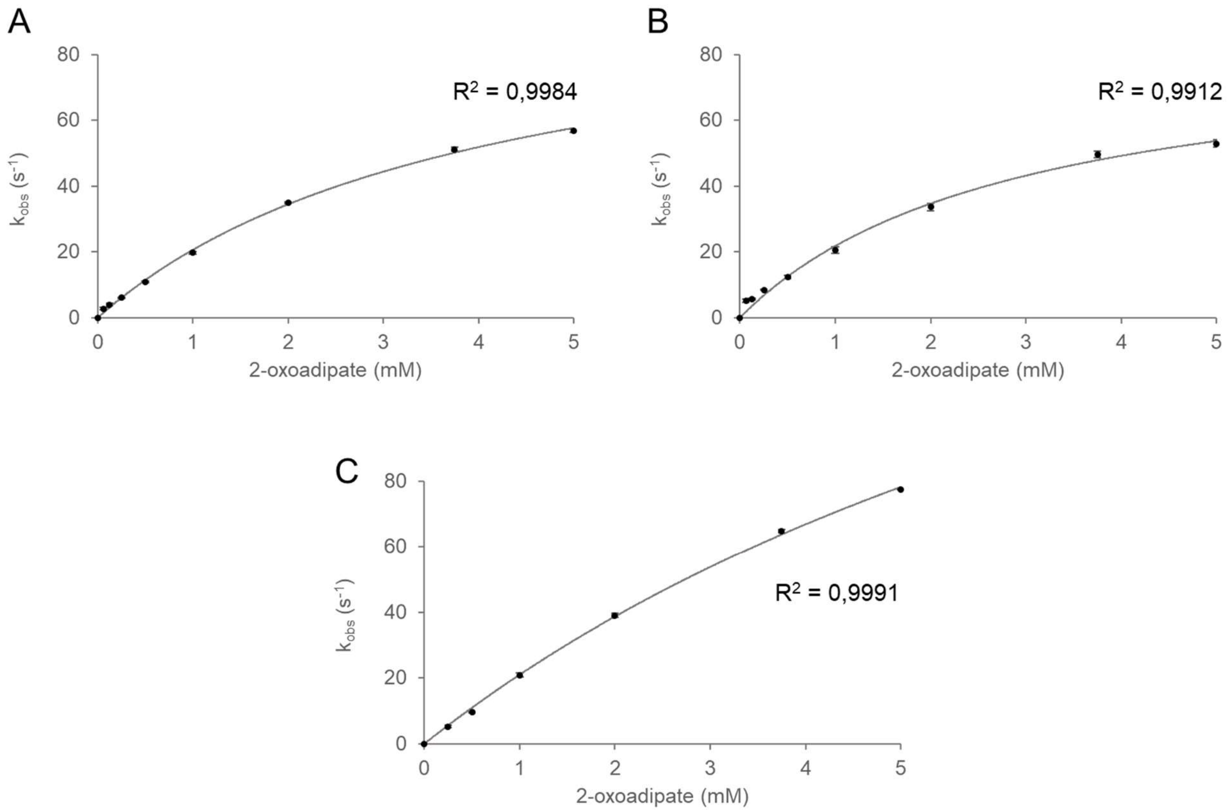

Figure S7. Kinetics of the improved variants with 2-oxoadipate. A, kinetics of Co1 variant; B, kinetics of Co2 variant; and C, kinetics of Co3 variant. Reactions were carried out at 25°C in 50 mM phosphate buffer, pH 8, containing 0.25 mM NADH. Results are means ± standard deviation values of triplicate assays.

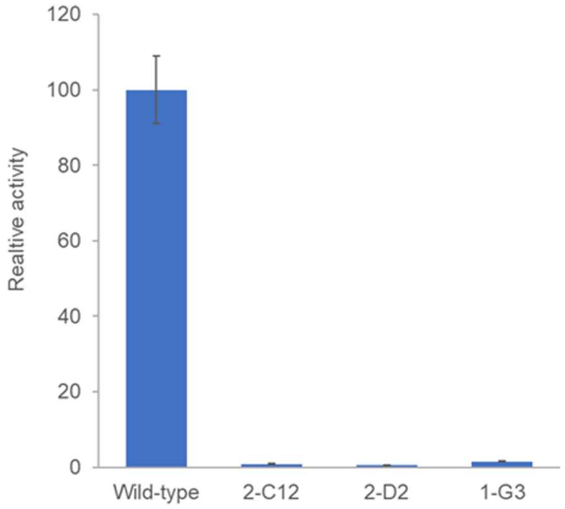

Figure S8. Activity of the improved variants Co1 (A2T/V11K/A206V/A214V/E304K), Co2 (A2T/V11K/A206V/A214D/K307I) and Co3 (A2T/V11K/A206V/A214V/V312I) towards 2-oxoglutarate. Reactions were carried out at 25°C in 50 mM phosphate buffer, pH 8 containing 0.5 mM 2-oxoglutarate, 0.25 mM NADH and appropriate aliquot of culture lysate. Results are means ± 95% confidence limits of replicate assays. The activity of the variants was normalized to the activity of the wild-type Hgdh.
